# CRISPR/Cas9-based genome-wide screening for deubiquitinase subfamily identifies USP1 regulating MAST1-driven cisplatin-resistance in cancer cells

**DOI:** 10.7150/thno.72826

**Published:** 2022-08-08

**Authors:** Apoorvi Tyagi, Kamini Kaushal, Arun Pandian Chandrasekaran, Neha Sarodaya, Soumyadip Das, Chang-Hwan Park, Seok-Ho Hong, Kye-Seong Kim, Suresh Ramakrishna

**Affiliations:** 1 Graduate School of Biomedical Science and Engineering, Hanyang University, Seoul, South Korea.; 2 Department of Internal Medicine, School of Medicine, Kangwon National University, Chuncheon, South Korea.; 3 College of Medicine, Hanyang University, Seoul, South Korea.

**Keywords:** Apoptosis, clinical tumor samples, DNA damage, drug resistance, kinase inhibitor, DUB inhibitor, ubiquitin proteasome system

## Abstract

**Background:** Cisplatin is one of the frontline anticancer agents. However, development of cisplatin-resistance limits the therapeutic efficacy of cisplatin-based treatment. The expression of microtubule-associated serine/threonine kinase 1 (MAST1) is a primary factor driving cisplatin-resistance in cancers by rewiring the MEK pathway. However, the mechanisms responsible for MAST1 regulation in conferring drug resistance is unknown.

**Methods:** We implemented a CRISPR/Cas9-based, genome-wide, dual screening system to identify deubiquitinating enzymes (DUBs) that govern cisplatin resistance and regulate MAST1 protein level. We analyzed K48- and K63-linked polyubiquitination of MAST1 protein and mapped the interacting domain between USP1 and MAST1 by immunoprecipitation assay. The deubiquitinating effect of USP1 on MAST1 protein was validated using rescue experiments, *in vitro* deubiquitination assay, immunoprecipitation assays, and half-life analysis. Furthermore, USP1-knockout A549 lung cancer cells were generated to validate the deubiquitinating activity of USP1 on MAST1 abundance. The USP1-MAST1 correlation was evaluated using bioinformatics tool and in different human clinical tissues. The potential role of USP1 in regulating MAST1-mediated cisplatin resistance was confirmed using a series of *in vitro* and *in vivo* experiments. Finally, the clinical relevance of the USP1-MAST1 axis was validated by application of small-molecule inhibitors in a lung cancer xenograft model in NSG mice.

**Results:** The CRISPR/Cas9-based dual screening system identified USP1 as a novel deubiquitinase that interacts, stabilizes, and extends the half-life of MAST1 by preventing its K48-linked polyubiquitination. The expression analysis across human clinical tissues revealed a positive correlation between USP1 and MAST1. USP1 promotes MAST1-mediated MEK1 activation as an underlying mechanism that contributes to cisplatin-resistance in cancers. Loss of USP1 led to attenuation of MAST1-mediated cisplatin-resistance both *in vitro* and *in vivo*. The combined pharmacological inhibition of USP1 and MAST1 using small-molecule inhibitors further abrogated MAST1 level and synergistically enhanced cisplatin efficacy in a mouse xenograft model.

**Conclusions:** Overall, our study highlights the role of USP1 in the development of cisplatin resistance and uncovers the regulatory mechanism of MAST1-mediated cisplatin resistance in cancers. Co-treatment with USP1 and MAST1 inhibitors abrogated tumor growth and synergistically enhanced cisplatin efficacy, suggesting a novel alternative combinatorial therapeutic strategy that could further improve MAST1-based therapy in patients with cisplatin-resistant tumors.

## Introduction

Platinum-based chemotherapy is a treatment widely used to treat several cancers 1, 2. Cisplatin is the first and most frequently used platinum-based drug, making it the frontline anticancer agent for treating a broad spectrum of cancers. Cisplatin eradicates cancer cells by crosslinking with DNA, which interferes with DNA synthesis and repair mechanisms and subsequently activates apoptotic pathways 3. Cisplatin has shown notable initial therapeutic success; however, many patients develop cisplatin-resistant tumor recurrence, which is a major limitation of cisplatin-based chemotherapy. Several processes account for cisplatin resistance, including increased drug efflux, DNA-adduct repair, and activation of pro-survival and inactivation of pro-apoptotic signaling pathways 3, 4.

Various signaling factors, such as MRP1, ATPase7A/7B/11B, and ERCC1, participate in the pre-target and on-target cisplatin-resistance mechanisms that allow tumor cells to elude cisplatin cytotoxicity 4. Several protein kinases, such as MKP-1 5, SRPK1 6, and RSK 7, have been identified as potential targets to combat cisplatin-resistance in different cancer types. Recent research has revealed that microtubule-associated serine/threonine-protein kinase 1 (MAST1) plays an essential role in driving post-target cisplatin-resistance mechanisms in many cancers, including head and neck, lung, and ovarian cancers 8. MAST1 is a protein kinase of the microtubule-associated serine/threonine kinase family 9 and functions as a scaffold molecule to link the dystrophin/utrophin complex with microfilaments via syntropin 10. Cisplatin inhibits the MAPK pathway by dissociating cRaf from the MEK1 complex, which upregulates the level of the pro-apoptotic factor BIM 8. MAST1 promotes pro-survival signaling by triggering MEK1 reactivation in a cRaf-independent manner, which produces cisplatin-resistance in human cancers 8.

The recurrent rearrangement and overexpression of MAST1 and MAST2 gene fusions reportedly have proliferative effects in breast cancer 11. Moreover, the high expression of MAST1 correlates positively with cisplatin-resistance in primary tumors acquired from patients who received cisplatin-based therapy. Treatment with lestaurtinib, a MAST1 inhibitor, effectively inhibits MAST1 kinase activity and reverses cisplatin-resistance in mouse models 8, 12, 13. The relative endogenous expression level of MAST1 is the main determinant in the development of cisplatin-resistance in various cancers; therefore, regulation of MAST1 protein abundance through the ubiquitin proteasomal system is critical. The E3 ligase carboxy-terminus of Hsc70 interacting protein (CHIP) binds and ubiquitinates the MAST1 protein to mark it for rapid degradation 12. However, the reversal of MAST1 ubiquitination by deubiquitinating enzymes (DUBs) which might also play an equally critical role in MAST1 stabilization and its association with cisplatin-resistance is still not explored.

Ubiquitin-specific proteases (USPs) are the largest subfamily of DUBs, which cleave ubiquitin moieties from their protein substrates and regulate multiple cellular processes, including cancer progression and chemoresistance 14-17. USP1 has been implicated in the DNA damage response and platinum resistance in different cancers 15, 18. Previous reports indicate that USP1 inhibition using small-molecule inhibitors such as pimozide effectively sensitizes cisplatin-resistant cancer cells 15, 19, 20.

In this study, we applied our recently developed CRISPR-based single-guide RNA (sgRNA) library targeting DUBs 21-23 to identify novel DUBs that might cause cisplatin-resistance in cancers. In parallel, we performed genome-wide screening for DUBs that regulate MAST1 protein abundance in cancer cells. Our dual screening approach identified USP1 as a protein regulator of MAST1 that confers cisplatin-resistance via MAST1-mediated activation of the MAPK pathway. We further demonstrated that a combinatorial treatment with small molecules targeting USP1 and MAST1 sensitized tumors to cisplatin treatment in a mouse xenograft model. Thus, we envision that the new targets regulating MAST1 protein abundance could help to reverse cisplatin-resistance in human cancers.

## Methods

### Cell culture

HEK293 (KCLB: 21573), HeLa (KCLB: 10002), and A549 (KCLB: 10185) cells were purchased from the Korean Cell Line Bank (Seoul, South Korea) and maintained in DMEM (Gibco BRL, Rockville, MD, USA) supplemented with 10% fetal bovine serum (FBS; Gibco) and 1% penicillin and streptomycin (Gibco) at 37 °C in a humidified atmosphere with 5% CO_2_. The cells were passaged every 2-3 days, depending on cell confluence.

### Cisplatin-resistant A549 and HeLa cells

Cisplatin-resistant (cis^R^) variants of A549 and HeLa cells were derived from their respective parental cell lines by gradual exposure to cisplatin (Sigma-Aldrich, UK). Briefly, the A549 and HeLa cells were seeded at a density of 1 x 10^6^ and subjected by stepwise increases to 16 μg of cisplatin/mL and 20 μg of cisplatin/mL, respectively, as described previously 24, 25. The cis^R^ cell lines were grown as monolayer cultures in the presence of cisplatin-containing DMEM supplemented with 10% FBS (Gibco) and 1% penicillin and streptomycin (Gibco) at 37 °C in a humidified atmosphere with 5% CO_2_.

### Plasmids, sgRNAs, and shRNAs

The full length human MAST1 gene was amplified from cDNA and cloned into pCDNA3-6XMyc-vector using BamHI and XbaI restriction sites. Flag-tagged USP1, USP1CS, and UAF1 were kindly provided by Prof. Yongzhong Liu (Shanghai Jiaotong University School of Medicine, China). A vector encoding HA-tagged ubiquitin (Cat. no. 18712), HA-tagged Lys(K) 48-ubiquitin (Cat. no. 17605), and HA-tagged Lys(K) 63-ubiquitin (Cat. no. 17606) were purchased from Addgene. The USP1 truncated mutants (UTMs): UTM1 (1-400 aa), UTM2 (401-785 aa), and UTM3 (201-785 aa) were cloned into the pCS4-3XFlag-vector using XhoI and XbaI restriction sites. The Cdh1 truncated mutants (CTMs): CTM1 (1-155 aa) and CTM2 (156-496 aa) were cloned into the pCS4-3XFlag-vector using EcoRI and XbaI restriction sites. For MAST1 truncated mutants (MTMs): MTM1 (1-832 aa), MTM2 (833-1570 aa), and MTM3 (1118-1465 aa), pCDNA3-6XMyc-vector digested with BamHI and XbaI restriction enzymes was used. All constructs were confirmed by DNA sequencing.

To screen potential DUB candidates, a plasmid encoding Cas9-2a-mRFP-2a-PAC (puromycin N-acetyl-transferase puromycin resistance gene) and a plasmid encoding sgRNA were purchased from Toolgen (Seoul, South Korea). The sgRNA target sequences were designed using a public tool (www.broadinstitute.org) and cloned into the vectors as described previously 26. Briefly, oligonucleotides containing each target sequence were synthesized (Bioneer, Seoul, South Korea), and T4 polynucleotide kinase was used to add terminal phosphates to the annealed oligonucleotides (Bio-Rad, CA, USA). The vector was digested using *BsaI* restriction enzyme and ligated with the annealed oligonucleotides. For short hairpin RNA (shRNA) generation, lentiviral vector constructs and packaging plasmids were kindly provided by Prof. Chung Hee Yong (Hanyang University, Seoul, South Korea). The target sequences for the sgRNAs individually targeting *USP1*, *UAF1*, and *Cdh1* genes and shRNA for *USP1* depletion are listed in Tables S1 and S2, respectively.

### Transfection and transduction

For transient transfection, HEK293 cells were transfected with plasmids using polyethyleneimine (Polysciences, Warrington, PA, USA) according to the manufacturer's protocol. HeLa, HeLa-cis^R^, and A549 cells were transfected with Lipofectamine 3000 (Cat. no. L3000001, Thermo Fisher Scientific).

USP1 lentivirus and shRNA targeting USP1 lentivirus were produced by co-transfecting constructs along with lentiviral packaging plasmids (pLP1, pLP2, and pLP-VSVG) into HEK293 cells in a 4:1:1:1 ratio. Cell supernatants were harvested 48 h post-transfection and were either used to infect cells or stored at -80 °C. Cells were infected at a low confluence (20%) for 6 h with lentiviral supernatants diluted 1:1 with normal culture medium in the presence of 10 ng/mL of polybrene (Sigma Aldrich) to obtain stable A549 and HeLa cell lines expressing Flag-USP1 or shRNA targeting USP1, respectively. After 48 h of infection, the cells were placed under puromycin selection for 2 days and then passaged before use.

### Antibodies and reagents

Mouse monoclonal antibodies against Flag (Anti-DDDDK-tag, M185-3L, 1: 1,000) were purchased from MBL Life Science, and phospho-Histone H2AX (Ser139) (Merck, 05-636) was purchased from Millipore. Mouse monoclonal antibodies against MAST1 (sc-373845, 1:50), UAF1 (also known as WDR48) (sc-514473; 1:500), H2AX (sc-517336; 1:1,000), MEK1 (sc-219), c-Myc (SC-40, 1:1,000), ubiquitin (sc-8017, 1:1,000), HA (sc-7392, 1:1,000), β-actin (sc-47778, 1:1,000), BIM (sc-374358, 1:1,000), USP9X (sc-365353, 1:1,000), GAPDH (sc-32233, 1:1,000), and normal mouse IgG (sc-2025, 1:1,000) were purchased from Santa Cruz Biotech. Rabbit polyclonal antibodies against MAST1 (13306-1-AP, 1:500, Proteintech; CSB-PA897529LA01HU, 1:100, Cusabio; CSB-PA013511GA01HU, 1:500, Cusabio), USP1 (14346-1-AP, 1: 2,000; Proteintech), ERK1/2 (CST, 9102, 1: 1,000; Cell Signaling Technology), phospho-ERK1/2 (CST, 9106, 1: 1,000; Cell Signaling Technology), phospho-MEK1 (S221) (Cat no. 9121, 1: 1,000, Cell Signaling Technology), BIM (ab15184, 1:25, Abcam), cleaved PARP (D64E10, 1: 1,000, Cell Signaling Technology), and 488/594-conjugated secondary antibodies (Cat. no. A21207 and Cat. no. A21203, 1:200; Life Technologies) were used. Protein A/G Plus agarose beads (sc-2003, Santa Cruz Biotech), protease inhibitor cocktail (Cat. no. 11836153001, Roche), IP lysis buffer (Cat. no. 87787; Thermo Fisher), cell lysis buffer (Cat. no. R2002, Biosesang), protein 5X sample buffer (Cat. no. EBA-1052, Elpis Biotech), protein translation inhibitor cycloheximide (CHX; Cat. no. 239765, Merck), proteasomal inhibitor MG132 (Cat. no. S2619, Selleckchem), puromycin (Cat. no. 12122530, Gibco), cisplatin (Cat no. P4394, Sigma-Aldrich), pimozide (Cat no. P1793, Sigma-Aldrich), lestaurtinib (Cat no. 3395, Tocris), DUB inhibitor, PR-619 (ab144641, Abcam), ubiquitin activating enzyme inhibitor, MLN7243 (also called TAK243, Cat no. HY-100487, MedChemExpress) and DAPI (Cat. no. H-1200, Vector Laboratories) were also used.

### Cell proliferation assay

For primary screening of DUBs conferring cisplatin-resistance, as shown by a cell viability assay, cisplatin-resistant HeLa cells were co-transfected with sgRNA targeting 50 DUBs and a Cas9 plasmid with the puromycin resistance gene. On the following day, the transfected cells were subjected to puromycin (2 µg/mL) selection and then re-seeded into 96-well plates for the assay. The cells were then treated with either vehicle (PBS) or cisplatin (5 μg/mL) for 48 h. Next, 10 μL of CCK-8 assay reagent (Dojindo Molecular Technologies, MD, USA) was added to each well, and absorbance was measured at 450 nm using a spectrophotometer (Bio-Rad Laboratories, Inc., Korea). The concentration of cisplatin used for A549 and cisplatin-resistant A549 is 2 μg/mL and 5 μg/mL, respectively.

### T7 endonuclease 1 assay

Genomic DNA was isolated using DNeasy Blood & Tissue kits (Promega, Madison, WI, USA) according to the manufacturer's protocol. The region of DNA containing the nuclease target site was PCR-amplified using hemi-nested or nested primers. Amplicons were denatured by heating and annealed to form heteroduplex DNA, which was then treated with 5 units of T7E1 (New England Biolabs, MA, USA) for 15 to 20 min at 37 °C, followed by 2% agarose gel electrophoresis. Mutation frequencies were calculated based on band intensity using ImageJ software and the following equation: mutation frequency (%) = 100 × (1 - [1 - fraction cleaved]^1/2^), where the fraction cleaved was the total relative density of the cleavage bands divided by the sum of the relative density of the cleaved and uncut bands. The oligonucleotide sequences used to obtain the PCR amplicons from on-target and off-target genes for the T7E1 assay are listed in Table S3 and Table S4, respectively. The amplicon sizes of the *USP1, Cdh1, UAF1*, and off-target genes and their expected cleavage sizes after the T7E1 assay are summarized in Table S5.

### Real-time PCR

Total RNA was isolated using Trizol reagent (Favorgen, Kaohsiung, Taiwan). RNA pellets were resuspended in 30 μl of nuclease-free water, and the RNA concentration was measured. Next, 500 ng of total RNA was reverse transcribed using a SuperScript III First-Strand Synthesis System (Life Technologies, USA) with an oligo-dT primer according to the manufacturer's protocol. Quantitative PCR was performed in triplicate using Fast SYBR Green I Master Mix (Life Technologies) and a Step One Plus Real-Time PCR System (Life Technologies). The USP1-targeting primers (5′- ATACTGAAGCTGAACGAAGTC-3′ and 5′-GATCTTGGAAAGTCCACCAC-3′) and MAST1-targeting primers (5′-TCTCTGGACCGCGCTTTCTA-3′ and 5′-TGAGGCTTTTCCGATTACTGGT-3′) were used with the loading control GAPDH-targeting primers (5′-CATGTTCGTCATGGGTGTGAACCA-3′ and 5′-AGTGATGGCATGGACTGTGGTCAT-3′).

### Generation of a *USP1*-knockout cell line using CRISPR/Cas9

A549 cells were co-transfected with a plasmid encoding Cas9 and sgRNA1 targeting *USP1* or scrambled sgRNA (mock control) at a 1:2 ratio using Lipofectamine 3000 Reagent (Thermo Fisher Scientific, MA, USA) according to the manufacturer's instructions. The next day, cells were selected using puromycin (2 µg/mL) for 2 days. The selected cells were then seeded into 96-well plates at an average density of 25 cells/plate. After 15 days, each well was microscopically evaluated, and round single cell-derived colonies were selected. The selected colonies were trypsinized and reseeded into 24-well cell culture plates. A small portion of the cells was screened for *USP1* gene disruption using the T7E1 assay. T7E1-positive clones were expanded and stored in a liquid nitrogen tank for later use. The USP1 and MAST1 mRNA and protein levels in the control and USP1 knockout groups were determined by RT-PCR and Western blotting.

### Immunoprecipitation

Cells were harvested 48 h post-transfection and lysed for 20 min in IP lysis buffer (25 mM Tris-HCl (pH 7.4), 150 mM sodium chloride, 1 mM EDTA, 1% NP-40, 5% glycerol, 1 mM PMSF, and protease inhibitor cocktail), and the amount of protein was estimated using Bradford reagent. Then, 2-3 mg of cell lysates was immunoprecipitated with the respective antibodies at 4 °C overnight and incubated with 25 μL of protein agarose beads at 4 °C for 3 h. Before loading the samples on SDS-PAGE gels, the beads were washed with lysis buffer and eluted in 2X SDS sample loading buffer (5X SDS sample loading buffer containing 4% SDS, 20% glycerol, 10% 2-mercaptoethanol, 0.004% bromophenol blue, and 0.125 M Tris-HCl [pH 6]). The samples were then boiled at 95 °C-100 °C for 5 min and analyzed by Western blotting. Mouse IgG (ab-99697, 1: 10,000; Abcam) and rabbit IgG (CST- 58802S, 1: 10,000; Cell Signaling Technology) light chain-specific secondary antibody was used to prevent interference from heavy and light immunoglobulin chains in the binding assay.

### Tandem ubiquitin-binding entities assay

The ubiquitination status of MAST1 protein was determined using a tandem ubiquitin binding entities (TUBEs) assay (Cat. no. UM402, LifeSensors, PA, USA) as previously described 27, 28. The mock control and USP1-KO1 and USP1-KO2 A549 cells were pretreated with the proteasome inhibitor MG132 (10 μM/mL) for 6 h before harvesting. The harvested cells were lysed in IP lysis buffer containing 150 mM sodium chloride, 1% Triton X-100, 25 mM Tris (pH 7.5), 1 mM EDTA, 10% glycerol, and protease inhibitor cocktail. The whole-cell protein extracts were incubated with 20 µL of ubiquitin affinity matrices-TUBE2 at 4 °C for 3 h with rotation. The beads were washed three times with IP lysis buffer and eluted in 30 µL 2X SDS sample loading buffer (5X SDS sample loading buffer contains 4% SDS, 20% glycerol, 10% 2-mercaptoethanol, 0.004% bromophenol blue, 0.125 M Tris-HCl (pH 6.8)) and boiled at 95 °C-100 °C for 5 min. The samples were then loaded onto SDS-PAGE gels and analyzed by Western blotting.

To assess the deubiquitinating effect of USP1 on MAST1 ubiquitination, *in vitro* deubiquitination assay was performed as previously described 27, 28. Before harvesting, the cells were treated with MG132 (10 µM/mL for 6 h) to accumulate polyubiquitinated MAST1 proteins. The cells were lysed using IP lysis buffer and then incubated with TUBE2 beads at 4 °C for 3 h. The beads were washed three times with IP lysis buffer and two times with ubiquitination buffer (50 mM Tris-HCl (pH 8.0), 10 mM MgCl_2_, 0.2 mM CaCl_2_, and 1 mM DTT) along with protease inhibitor cocktail, followed by incubation with 1.5 μg of recombinant USP1 protein (rUSP1) (catalog No. E-568-050; R&D Systems) at 37 °C for 1 h. The rUSP1-treated samples were eluted with 30 μL of 2X SDS sample loading buffer and boiled for 5 min before subjecting to Western blot analysis.

### Deubiquitination assay

The DUB activity of USP1 on endogenous and exogenous MAST1 protein was determined in HeLa and HEK293 cells, respectively. After 48 h, cells were treated with MG132 (5 µM/mL for 6 h) and harvested. The cells were lysed for 20 min in denaturing lysis buffer containing 150 mM sodium chloride, 1% Triton X-100, 1% sodium deoxycholate, 1% SDS, 50 mM Tris-HCl (pH 7.4), 2 mM EDTA, 1 mM PMSF, and protease inhibitor cocktail. Then, 2-3 mg of cell lysates was immunoprecipitated with the respective antibodies at 4 °C overnight and incubated with 25 μL of protein agarose beads for 2-3 h at 4 °C. The beads were then washed with lysis buffer and eluted in 2X SDS sample loading buffer (5X SDS sample loading buffer contains 4% SDS, 20% glycerol, 10% 2-mercaptoethanol, 0.004% bromophenol blue, and 0.125 M Tris-HCl (pH 6.8)) and boiled at 95 °C-100 °C for 5 min. The samples were then loaded onto SDS-PAGE gels and analyzed by Western blotting using ubiquitin and HA-antibodies. To confirm the specificity of MAST1 ubiquitination and to avoid non-specific binding of polyubiquitin molecules to MAST1 protein, we washed the protein-bound beads with lysis buffer containing 300 mM NaCl for the experiments represented in Figure 4J and Figure S6A as previously described 29.

### Immunofluorescence

HeLa-cis^R^ and A549 cells were grown on glass coverslips and incubated at 37 °C in a humidified atmosphere with 5% CO_2_. After a wash with Phosphate-Buffered Saline (PBS, Gibco) the cells were fixed for 15 min using 4% paraformaldehyde (PFA, Biosesang) and permeabilized in PBS containing 0.1% Triton X for 5 min. The cells were then thoroughly washed in PBS and blocked with 5% bovine serum albumin, followed by incubation with appropriate primary antibodies overnight at 4 °C. The next day, the cells were washed and incubated with the appropriate Alexa Fluor 488/594-conjugated secondary antibodies for 1 h. The cells were then incubated with DAPI and mounted using VectaShield (Vector Laboratories, CA, USA). The cells were visualized, and images were produced using a Leica fluorescence microscope (Leica, DM 5000B; Leica CTR 5000; Wetzlar, Germany).

### Duolink proximity ligation assay

The interaction between USP1 and MAST1 was observed using a Duolink *in situ* proximity ligation assay (PLA) kit (Cat. no. DUO92101, Sigma Aldrich). A549 cells were fixed in 4% PFA for 10 min at room temperature and then blocked with blocking solution. The cells were treated with primary antibodies targeting USP1 and MAST1 for 1 h at 37 °C, followed by incubation with PLA probes for 1 h at 37 °C in a humidified chamber. After three washes, ligation-ligase solution was added, and the cells were incubated for 30 min at 37 °C. The slides were incubated for 100 min in an amplified polymerase solution at 37 °C in the dark. Finally, the cells were stained with mounting medium containing DAPI. A Leica fluorescence microscope was used to capture the fluorescence images (Leica, DM 5000B; Leica CTR 5000; Wetzlar, Germany).

### Immunohistochemistry

To perform correlation analysis with clinical tissue samples, we purchased tissue microarray slides of breast (n = 21), colon (n = 32), and lung (n = 32) cancer tissues from ISU Abxis (Gyeonggi-do, South Korea). Formalin-fixed, paraffin-embedded (FFPE) tissue samples were processed and incubated with USP1 or MAST1 antibody according to the supplier's protocol. These samples were counterstained with hematoxylin, dehydrated, and mounted.

The tumor-tissue samples obtained from the mice were fixed with 4% PFA and embedded in paraffin. FFPE tissues were then sectioned at a thickness of 5 µm and stained with USP1, MAST1, BIM, and cleaved PARP antibodies following the manufacturers' recommendations. All images were produced using a Leica DM5000 B microscope (Leica, Germany).

### IHC assessment

For the assessment of USP1 and MAST1 expression in microarray slides of breast (n = 21), colon (n = 32), and lung (n = 32) cancer tissues, IHC was performed and the staining intensity was classified as low, moderate and intense according the degree of staining. To interpret the results, the raw data was binarised for statistical analysis where least expression intensity for USP1 and MAST1 was categorized as negative group. The moderate and high expression intensity for USP1 and MAST1 was categorized as positive group. The relationship between the different tumors and the protein expression level of USP1 and MAST1 was analyzed using Chi-square test. The correlation between USP1 and MAST1 protein staining intensity in different tumors were estimated by histochemical scoring (H-score). The H-score was determined by adding the results of multiplication of the percentages of cells with staining intensity ordinal values.

### Drug combination studies

The combination effect of lestaurtinib and pimozide on growth of cisplatin-resistant cells were analyzed using CompuSyn 1.0 software as described 30, 31. Briefly, the individual dose-effect of both lestaurtinib and pimozide was obtained by treating A549-cis^R^ and HeLa-cis^R^ with lestaurtinib and pimozide in the presence of a sublethal dose of cisplatin. The median effect dose (Dm) and linear correlation coefficient of the ME-plot (r) were analyzed. Optimal concentration ratios were obtained on the basis of Dm values, and serial dilutions were used to measure the combination index (CI). CI values less than 1, equal to 1, and greater than 1 indicated synergistic, additive, and antagonistic effects, respectively.

### Apoptosis assay

To estimate cellular apoptosis, the annexin V/7-AAD population was detected using a BD FACSCanto II flow cytometer (BD Biosciences, CA, USA). Briefly, A549 cells (mock control, USP1KO, USP1KO-reconstituted with USP1, and USP1KO-reconstituted with MAST1) were treated with either vehicle or cisplatin (2 µg/mL) for 48 h. The cells were then harvested and washed twice with PBS containing 10% FBS. The required cells were counted, and 5 µL of annexin-V and 7-AAD were added to cells prior to incubation for 15 min. The stained cells were resuspended in the binding buffer, and flow cytometry was performed within 1 h. For propidium iodide (PI) staining (BD Biosciences), the same cell groups were treated with vehicle or cisplatin for 48 h and then harvested, washed twice with ice-cold PBS containing 10% FBS, and fixed with ice-cold 70% ethanol until use. Then, the cells were resuspended in PI (50 µg/mL; Sigma) and RNase A (200 µg/mL, New England Biolabs, MA, USA) and subjected to a FACS analysis (BD FACSCanto II, BD Biosciences) to estimate the DNA content.

### Soft agar assay

Mock control, USP1-KO1 and USP1-KO2, USP1KO clones reconstituted with USP1, and USP1KO clones reconstituted with MAST1 were subjected to a colony formation assay. First, 1% agarose gel and 1X complete DMEM were mixed at a ratio of 1:1 and plated onto 35 mm culture dishes. The plates were then incubated overnight. The next day, cells resuspended in 0.75% agarose with DMEM (1:1 ratio) were seeded at a density of 1 × 10^4^ cells per well. The cells were treated with vehicle or the indicated drugs every other day for 14 days. Crystal violet dye (0.01%) diluted in 20% methanol was used to stain the anchorage-independent colonies, and they were counted using a light microscope (IX71, Olympus, Tokyo, Japan).

### Wound healing assay

Migration activity was analyzed using the wound healing assay. Mock control, USP1-KO1 and USP1-KO2, USP1KO clones reconstituted with USP1, and USP1KO clones reconstituted with MAST1 were cultured to near 90% confluence. Scratches were made in the monolayers with a sterile pipette tip in a definite array. The wounded cell layer was washed with PBS and incubated in medium containing indicated drugs at 37 °C with 5% CO_2_. Wound closure was compared between conditions at 0 h and 36 h using a light microscope and quantified using ImageJ software.

### Transwell cell invasion assay

Cellular invasion was assessed using 0.8 µm Transwell chambers coated with Matrigel for 1 h at 37 °C (Corning, NY, USA) according to the manufacturer's instructions. Briefly, mock control, USP1-KO1 and USP1-KO2, USP1KO clones reconstituted with USP1, and USP1KO clones reconstituted with MAST1 were seeded at a density of 3.0 × 10^4^ cells per well in 500 µL of serum-free DMEM in 24-well plates. Next, 750 µL of complete medium with the indicated drugs was added and incubated at 37 °C with 5% CO_2_. The following day, the cells on the top surface of the insert were scraped off, and the cells on the bottom surface were fixed with ice-cold methanol followed by crystal violet staining. The number of cells was counted using light microscopy, and the data are presented graphically.

### Cisplatin sensitivity assay

For the cisplatin sensitivity assay, 0.7 × 10^4^ cells were seeded in each well of 96-well plates and incubated at 37 °C with 5% CO_2_ overnight. Next day, the cells were treated with increasing concentrations of cisplatin along with pimozide (50 μM) or lestaurtinib (200 nM) or both for 48 h. Cell viability was estimated using a CCK-8 assay reagent kit, and IC_50_ was calculated using GraphPad software.

### Animal studies

Xenograft tumor experiments were performed in NSG mice (6 weeks old). The animal study was approved by the Institutional Animal Care and Use Committees of Hanyang University. All mice were housed in a temperature-controlled room under standard conditions (12h light/dark cycle and 55% relative humidity) with access to food and water *ad libitum*. Mock, USP1-KO1, USP1-KO1-reconstituted with USP1, and USP1-KO1-reconstituted with MAST1 A549 cells were prepared in DMEM: Matrigel (1:1) (BD Biosciences) and subcutaneously injected into the right flank of each mouse, followed by intraperitoneal (i.p.) injection of cisplatin (2 mg/kg) twice a week for 14-16 days. The tumors were harvested at each experimental endpoint and subjected to immunohistochemical analyses.

For the pharmacological studies, A549-cis^R^ cells (4 × 10^6^) were subcutaneously injected into the right flank of each mouse, and the tumors were allowed to reach 110-150 mm^3^. Cisplatin (5 mg/kg) and pimozide (10 mg/kg) were administered twice a week by i.p. injection, and lestaurtinib (20 mg/kg) was administered three times a week by subcutaneous injection. The tumors were harvested at the experimental endpoint, and images were taken. Tumor growth was recorded by measuring two perpendicular diameters (short axis and long axis), and tumor volume was calculated using the formula, V = D × d2 × 0.5, where D is the long axis, and d is the short axis of the tumor. For all animal studies, animals were randomly chosen.

### Statistical analysis

Statistical analysis and graphical presentation were performed using GraphPad Prism 9.0. No statistical method was used to predetermine the sample size. All results are presented as the means and standard deviations of at least three independent experiments (unless otherwise stated in the figure legends). Comparisons between two groups were analyzed using Student's *t*-test. Experiments involving three or more groups were analyzed by one-way or two-way analysis of variance (ANOVA) followed by Tukey's post hoc test.

## Results

### CRISPR/Cas9-based genome-scale screening for DUBs that confer cisplatin-resistance in cancers

**Figure 1:**
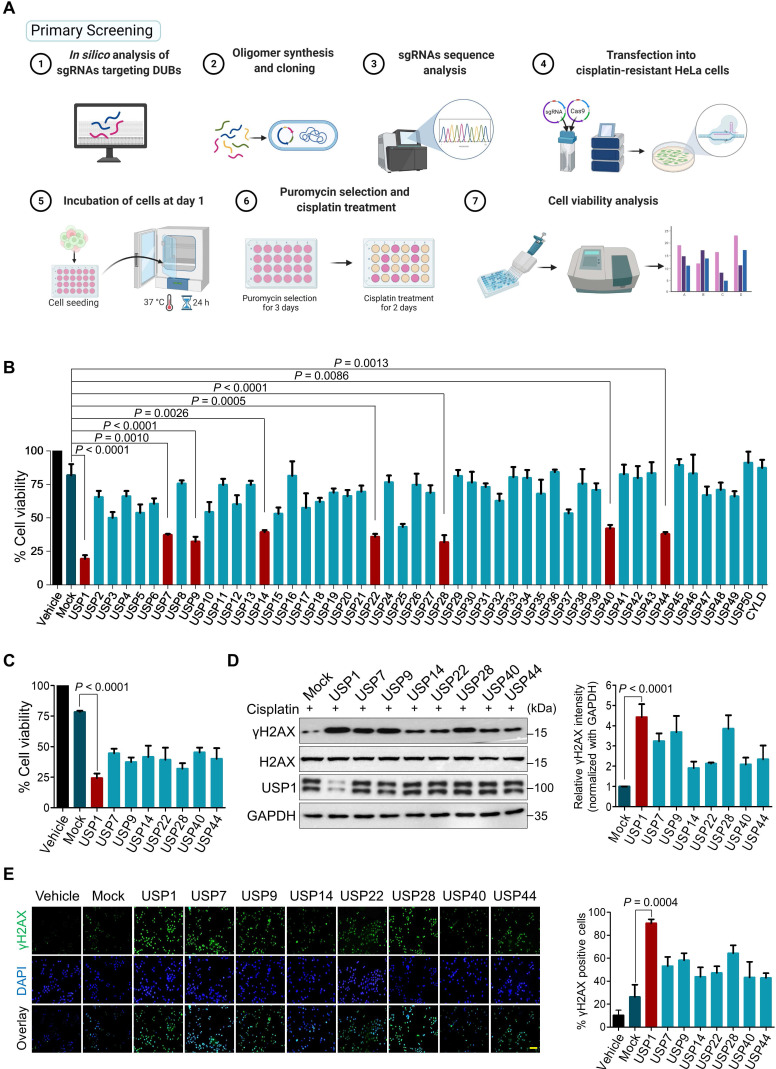
** CRISPR/Cas9-based genome-scale screening of USP subfamily proteins showing drug resistance to cisplatin treatment. (A)** Schematic illustration of primary screening using CRISPR/Cas9-based DUB knockout screening system and cisplatin treatment. Step 1: *In silico* analysis to design sgRNAs targeting entire USP subfamily genes with high cleavage efficiency and low off-target scores. Steps 2-3: sgRNA synthesis and cloning into U6 promoter-driven plasmid followed by sequence analysis. Steps 4-5: The sgRNA library targeting an entire set of genes encoding USPs was co-transfected with Cas9 into HeLa-cis^R^ cells and incubated for 24 h (day 1). Step 6: sgRNA-transfected cells were selected by puromycin (2 µg/mL) for 3 days (days 2-5). On day 9, the puromycin-selected HeLa-cis^R^ cells were re-seeded into 96-well plates at a density of 10,000 cells/well. The cells were cultured in vehicle or a sub-lethal dose of cisplatin for 48 h (days 10-12). Step 7: Cells were subjected to the cell viability assay using a CCK-8 kit. **(B)** The cisplatin-induced cell death from (A) was estimated using a cell viability assay and plotted as a bar graph. Vehicle-treated HeLa-cis^R^ cells served as the negative control, and cisplatin-treated HeLa-cis^R^ cells co-transfected with scrambled sgRNA and Cas9 served as the mock control. Data are presented as the mean and standard deviation of three independent experiments (n = 3). One-way ANOVA followed by Tukey's post hoc test was used with the indicated *P* values. **(C)** Cell viability of the putative DUB candidates. Data are presented as the mean and standard deviation of three independent experiments (n = 3). One-way ANOVA followed by Tukey's post hoc test was used with the indicated *P* values. **(D)** The HeLa-cis^R^ cells were transfected with sgRNAs targeting the putative candidates and then exposed to a sub-lethal dose of cisplatin to assess the cisplatin-induced DNA damage using γH2AX antibodies. H2AX and GAPDH were used as loading controls. The relative expression of γH2AX was quantified with ImageJ software (right panel). Data are presented as the mean and standard deviation of three independent experiments (n = 3). One-way ANOVA followed by Tukey's post hoc test was used with the indicated *P* values.** (E)** Immunofluorescence staining showing γH2AX expression in HeLa-cis^R^ cells transfected with sgRNAs targeting the indicated DUB candidates and exposed to vehicle or cisplatin. γH2AX-positive cells were quantified, and the results are represented as a bar graph (right panel). Data are presented as the mean and standard deviation of three independent experiments (n = 3). One-way ANOVA followed by Tukey's post hoc test was used with the indicated *P* values. Scale bar = 100 µm.

Our primary screening aimed to identify DUBs responsible for cisplatin-resistance in human cancers by implementing a loss-of-function-based sgRNA library targeting DUBs 21. An entire set of sgRNAs individually targeting USP subfamily genes was co-transfected with Cas9 into cisplatin-resistant HeLa (HeLa-cis^R^) cells. The transfected cells were then treated with a sub-lethal dose of cisplatin (5 μg/mL). The USP genes whose depletion increased the cytotoxicity of cisplatin were examined using a cell viability assay (Figure 1A). We identified sgRNAs targeting USP1, USP7, USP9, USP14, USP22, USP28, USP40, and USP44 as significant hits whose depletion increased the lethality of cisplatin (Figure 1B, C). Cisplatin causes cytotoxicity by crosslinking with DNA, which elevates the expression of γH2AX, a DNA damage marker 32. Among the USPs that increased the lethality of cisplatin, USP1 emerged as the lead hit whose depletion resulted in high cisplatin cytotoxicity (Figure 1C) with increased γH2AX expression (Figure 1D) and foci formation compared with the mock control and other putative DUB candidates (Figure 1E).

### Loss-of-function-based screening for DUBs regulating MAST1 protein level

**Figure 2:**
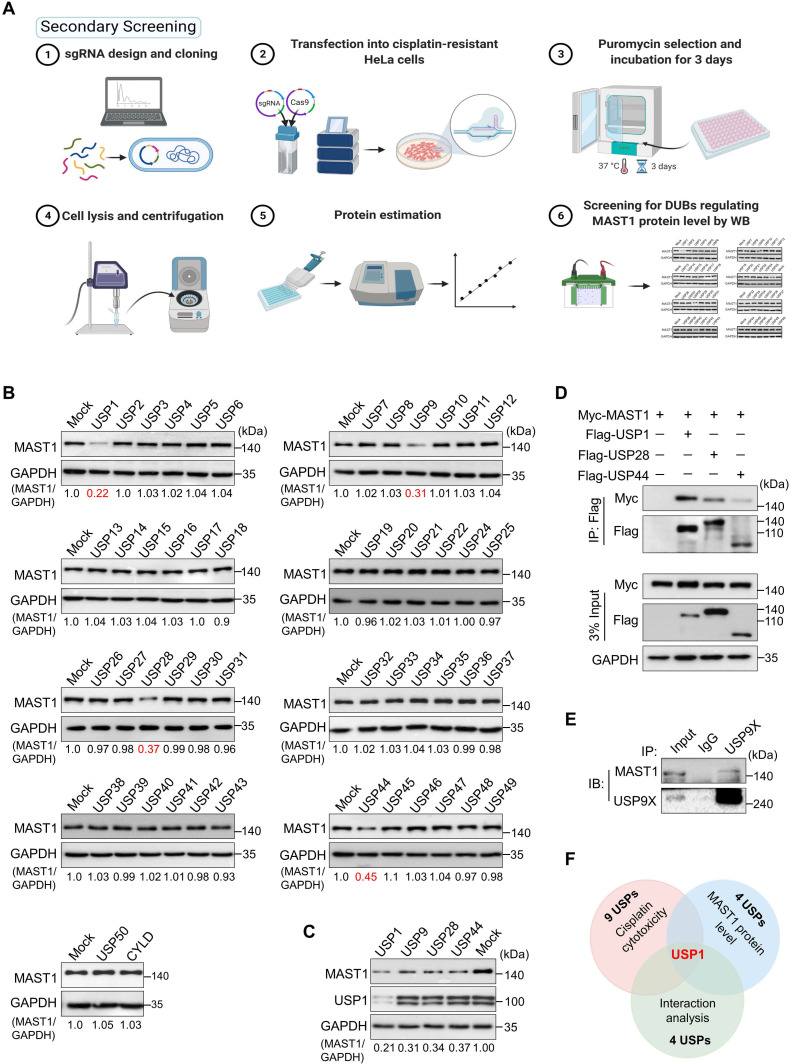
** DUB knockout library kit-based screening for USPs regulating MAST1 protein level by Western blot analysis. (A)** Schematic representation of secondary screening with a CRISPR/Cas9-based sgRNA library to find DUBs that regulate MAST1 protein level. Steps 1-2: The designed DUB knockout sgRNA library, which consists of an entire set of genes encoding USPs, was co-transfected with Cas9 into HeLa-cis^R^ cells (day 1). Step 3: The cells were placed under puromycin selection (2 µg/mL) and incubated for 3 days (days 2-5). Step 4: The transfected cells were harvested and lysed, and protein was isolated. Steps 5-6: Protein concentration was estimated by Bradford reagent, and equal concentrations of all DUBKO cell lysates were loaded on SDS-PAGE and screened for DUB candidates regulating endogenous expression pattern of MAST1 using Western blot (WB) analysis. **(B)** Equal protein concentrations from the cell lysates from (A) were subjected to Western blotting to determine the endogenous MAST1 protein level. For each blot, HeLa-cis^R^ cells co-transfected with scrambled sgRNA and Cas9 served as the mock control. GAPDH was used as a loading control. The protein band intensities were estimated using ImageJ software with reference to the GAPDH control for each individual sgRNA (MAST1/GAPDH) and presented below the blot. **(C)** The effects of the targeting the putative DUB candidates on the MAST1 protein level were estimated by Western blotting. The protein band intensities were estimated using ImageJ software with reference to the GAPDH control band for each individual sgRNA (MAST1/GAPDH) ) and presented below the blot. **(D)** The interactions between putative DUB candidates and MAST1 by co-immunoprecipitation analysis. Myc-MAST1 and DUBs (Flag-USP1, Flag-USP28, and Flag-USP44) were transfected into HEK293 cells. **(E)** The interaction between endogenous USP9X and MAST1 by co-immunoprecipitation analysis. Cell lysates were immunoprecipitated and immunoblotted with the indicated antibodies.** (F)** A Venn diagram showing the overlapping DUB candidate based on cisplatin cytotoxicity, loss-of-function effect on MAST1 protein level, and interaction analysis with MAST1.

Given that MAST1 is a potential therapeutic target to battle cisplatin-resistance in cancers, our secondary screening aimed to identify DUBs that regulate MAST1 protein abundance. To this end, we analyzed MAST1 protein level as we depleted DUBs in cisplatin-resistant HeLa cells using our previously generated CRISPR/Cas9-based sgRNA library targeting DUBs 21 (Figure 2A). We identified several putative DUBs, including USP1, USP9, USP28, and USP44, whose depletion decreased the MAST1 protein level (Figure 2B). Among those USPs, USP1 was the strongest candidate that produced the most significant reduction in MAST1 protein (Figure 2C). We further confirmed the interaction between putative USPs and MAST1 by co-immunoprecipitation. We observed that USP1 showed greater interaction intensity with MAST1 compared with other USPs (Figure 2D, E). Overall, USP1 emerged as the top-ranking DUB candidate from our primary screening based on cisplatin cytotoxicity, secondary screening based on MAST1 protein level, and interaction studies (Figure 2F).

### USP1 increases MAST1 protein level

**Figure 3:**
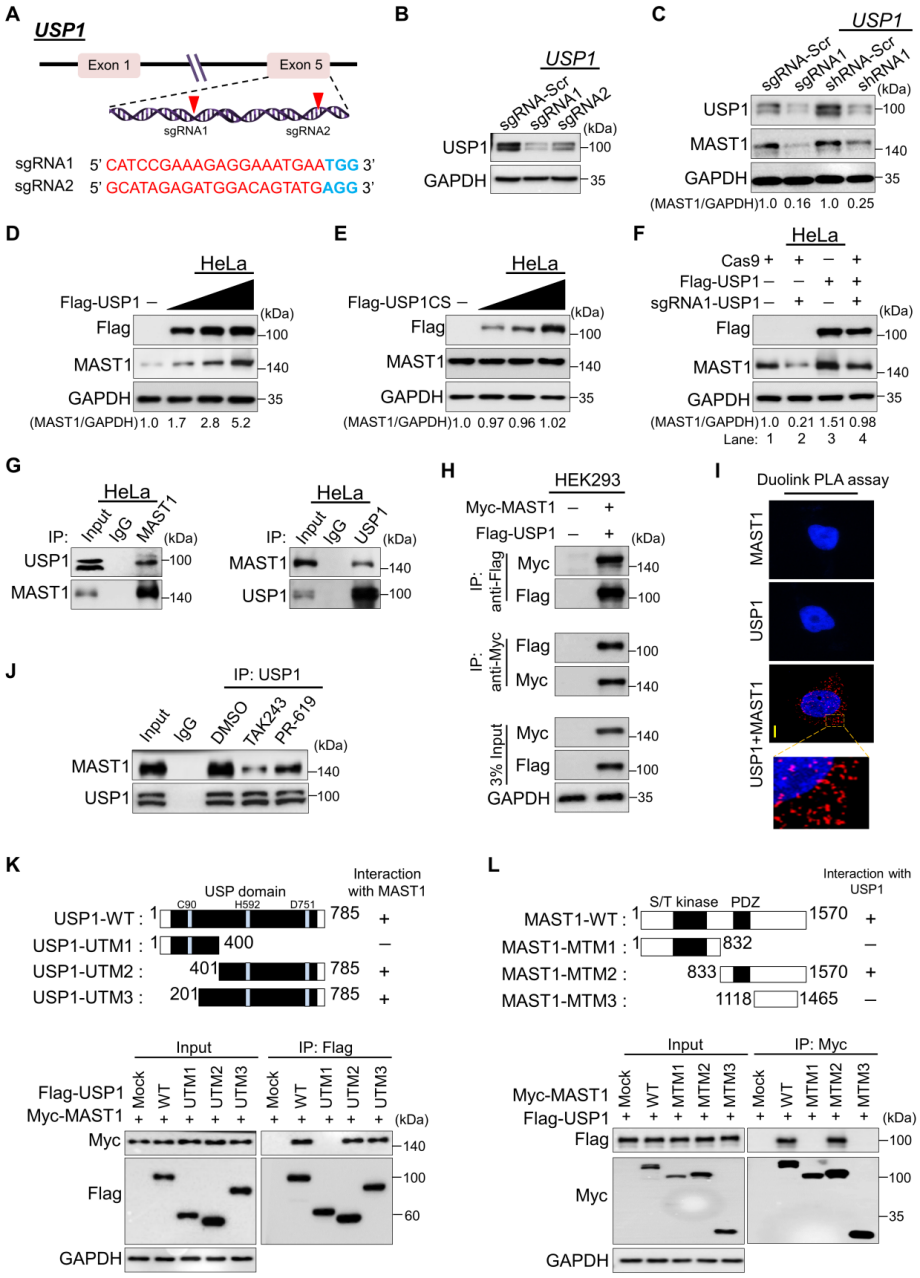
** USP1 interacts with and regulates the MAST1 protein. (A)** Schematic representation of the sgRNAs targeting exon 5 of the *USP1* gene. Red arrowheads indicate the positions of sgRNAs that target the top strand. sgRNA sequences are in red; PAM sequences are in bold blue font. **(B)** Validation of sgRNA efficiency targeting *USP1* by transient transfection of sgRNA1 and sgRNA2 into HeLa cells and immunoblotting with USP1 antibody. **(C)** HeLa cells were transfected with sgRNA1 and shRNA1 targeting *USP1*, and the endogenous protein levels of USP1 and MAST1 were checked by Western blotting. **(D)** HeLa cells were transfected with increasing concentrations of Flag-USP1 to check the endogenous MAST1 protein level. **(E)** HeLa cells were transfected with increasing concentrations of Flag-USP1CS to assess the endogenous MAST1 protein level. **(F)** The reconstitution effect of Flag-USP1 on endogenous MAST1 protein in USP1-depleted HeLa cells. The protein band intensities (Fig 3C-F) were estimated using ImageJ software with reference to the GAPDH control band for each individual sgRNA (MAST1/GAPDH) and presented below the blot. **(G)** Interactions between endogenous and **(H)** exogenous USP1 and MAST1 proteins were analyzed in HeLa cells and HEK293 cells, respectively. Cell lysates were immunoprecipitated and immunoblotted with the indicated antibodies. Protein expression was checked using Western blotting. GAPDH was used as a loading control. **(I)** HeLa cells were subjected to the Duolink PLA assay to analyze the interaction between USP1 and MAST1 using specific antibodies. *In situ* USP1-MAST1 interaction (PLA dots) was observed when USP1 and MAST1 were immunostained together but not when they were stained with individual antibodies. Scale bar: 10 µm. **(J)** HeLa cells were treated with either MLN7243 (10 µM) or PR-619 (20 µM) for 1 h before harvesting. Cell lysates were immunoprecipitated and immunoblotted with the indicated antibodies. **(K)** Schematic representation of full length USP1 (1-785 aa) encoding USP domain with catalytic triad residues (Cys, His, and Asp box) (represented as USP1-WT), N-terminus USP1 (1-400 aa) encoding catalytic Cys box (represented as USP1-UTM1), C-terminus USP1 (401-785 aa) encoding catalytic His and Asp box (represented as USP1-UTM2), and extended C-terminus USP1 (201-785 aa) encoding catalytic His and Asp box (represented as USP1-UTM3). Interactions between full length MAST1 and USP1 truncated mutants by co-immunoprecipitation and immunoblotting with the indicated antibodies (lower panel). **(L)** Schematic representation of full length MAST1 (1-1570 aa) encoding serine/threonine (S/T) kinase domain and PDZ domain (represented as MAST1-WT), N-terminus MAST1 (1-832 aa) encoding S/T kinase domain (represented as MAST1-MTM1), C-terminus MAST1 (833-1570 aa) encoding PDZ domain (represented as MAST1-MTM2), and C-terminus MAST1 (1118-1465 aa) lacking PDZ domain (represented as MAST1-MTM3). Interactions between full length USP1 and MAST1 truncated mutants were analyzed by co-immunoprecipitation and immunoblotting with the indicated antibodies (lower panel).

To validate that USP1 regulates the MAST1 protein level, we applied both sgRNA and short hairpin RNA (shRNA) systems to silence *USP1* gene expression. To this end, we designed sgRNA1 and sgRNA2, targeting exon 5 of the *USP1* gene (Figure 3A). The protein expression of USP1 was highly reduced by sgRNA1 compared to sgRNA2 (Figure 3B), which is in line with the high indel percentage observed with sgRNA1 (Figure S1A). Likewise, shRNA1 showed greater reduction of USP1 expression than shRNA2 (Figure S1B). Therefore, we used the highly efficient sgRNA1 (hereafter sgRNA) and shRNA1 (hereafter shRNA) to assess the effects of USP1 depletion on MAST1 protein level. HeLa cells transfected with the sgRNA or shRNA targeting *USP1* showed a substantial reduction in MAST1 protein level compared with the mock control (Figure 3C).

USP1 requires an allosteric activator, USP1-associated factor 1 (UAF1), which forms a stable heterodimeric complex with USP1 and enhances its deubiquitinating activity 33. Thus, we investigated the biological significance of UAF1 on MAST1 protein expression and MAST1-driven cisplatin resistance. We designed and validated two sgRNAs targeting *UAF1* and observed that sgRNA1 is more efficient than sgRNA2 (Figure S2A, B). Consistent with a previous report 33, decreased UAF1 level in *UAF1*-depleted cells was accompanied by reduced level of USP1 (Figure S2B). Moreover, the depletion of *UAF1* reduced MAST1 protein levels (Figure S2B) and subsequently increased the cytotoxicity of cisplatin in HeLa-cis^R^ cells (Figure S2C), suggesting that the UAF1 complex with USP1 is critical for MAST1-driven cisplatin resistance. Therefore, we co-transfected UAF1 along with USP1 wherever applicable (for brevity, we call the USP1/UAF1 complex simply USP1). The transfection of USP1 produced a dose-dependent increase in the level of endogenous MAST1 protein (Figure 3D), whereas the overexpression of catalytic mutant USP1 C90S (USP1CS) did not affect the MAST1 protein level (Figure 3E). Furthermore, the reduced expression of endogenous MAST1 protein in *USP1-*depleted cells was rescued by reconstitution with Flag-USP1 (Figure 3F, lane 4 vs. 2). Likewise, the upregulating effect of USP1 on MAST1 level was cross-confirmed in HEK293 cells (Figure S3A-C), demonstrating that USP1 is a protein regulator of MAST1.

### USP1 interacts with MAST1

To illustrate the mechanism of USP1-mediated MAST1 regulation, we first tested whether endogenous USP1 interacts with the MAST1 protein. Our co-immunoprecipitation analysis using specific antibodies against endogenous USP1 and MAST1 demonstrated that USP1 interacts with MAST1 protein and vice versa under physiological conditions (Figure 3G). Similarly, exogenous Flag-USP1 interacted with Myc-MAST1 and vice versa (Figure 3H). Additionally, we demonstrated the interaction between USP1 and MAST1 using a Duolink PLA assay. The *in situ* USP1-MAST1 interaction was confirmed by fluorescence signals (PLA dots) when USP1 and MAST1 were immunostained together, but no PLA dots were observed when the cells were stained with either the USP1 or MAST1 antibody alone (Figure 3I). Furthermore, to check whether the interaction between USP1 and MAST1 depends on the ubiquitination status of MAST1 protein, we treated cells with ubiquitin-activating enzyme inhibitor, MLN7243 and DUB inhibitor PR-619 followed by co-immunoprecipitation analysis. The treatment with ubiquitination inhibitor, MLN7243 and DUB inhibitor, PR-619 reduced the interaction between USP1 and MAST1 when compared to DMSO treated sample (Figure 3J), suggesting that their interaction is dependent on the ubiquitination status of MAST1 protein.

To further corroborate our finding that USP1 interacts with MAST1 protein, we sought to identify which USP1 regions are critically required for its interaction with MAST1, and *vice versa*. To this end, we generated three USP1 truncated mutants (UTMs), i.e., N-terminus USP1 (UTM1: 1-400 aa) having USP domain encoding catalytic Cysteine (Cys) box, C-terminus USP1 (UTM2: 401-785 aa) having USP domain encoding catalytic Histidine (His) box and Aspartic acid (Asp) box, and extended C-terminus USP1 (UTM3: 201-785 aa) having USP domain encoding catalytic His and Asp box (Figure 3K, upper panel). The co-immunoprecipitation assays showed that UTM2 and UTM3 interacted with full length MAST1, while UTM1 did not associate with MAST1 (Figure 3K, lower panel). On the other hand, we generated three MAST1 truncated mutants (MTMs), i.e., N-terminus MAST1 (MTM1: 1-832 aa) encoding serine/threonine (S/T) kinase domain, C-terminus MAST1 (MTM2: 833-1570 aa) encoding the PDZ domain, and C-terminus MAST1 (MTM3: 1118-1465 aa) lacking the PDZ domain (Figure 3L, upper panel). Binding assays showed that MTM2 with the PDZ domain interacted with full length USP1, while MTM1 and MTM3 did not associate with USP1 (Figure 3L, lower panel). Several reports suggested that the PDZ domain is essential for interaction with its binding partners 34-37. In line with previous reports, the PDZ domain in the C-terminus region of MAST1 was necessary to interact with USP1. Collectively, our data showed that USP1 regulates MAST1 stability through its interaction with MAST1 protein.

### E3 ligase Cdh1 interacts with and reduces the MAST1 protein level

**Figure 4:**
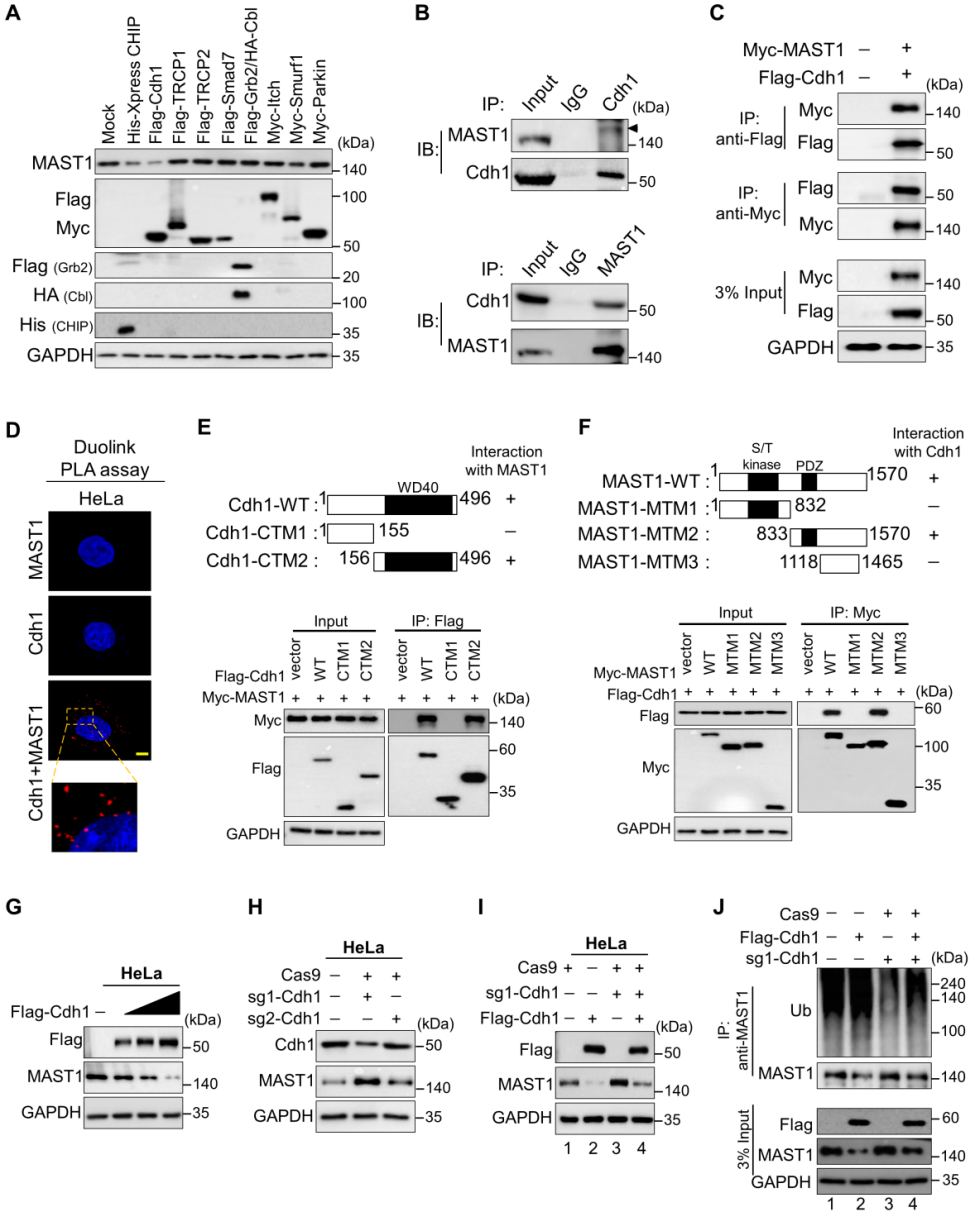
** The E3 ligase Cdh1 interacts with and downregulates MAST1 protein. (A)** HeLa cells were transfected with a panel of E3 ligases, and the expression of MAST1 protein was analyzed using Western blotting. **(B)** Interactions between endogenous and **(C)** exogenous Cdh1 and MAST1 proteins were analyzed in HeLa cells and HEK293 cells, respectively. Cell lysates were immunoprecipitated and immunoblotted with the indicated antibodies. Protein expression was checked using Western blotting. GAPDH was used as a loading control. **(D)** HeLa cells were subjected to the Duolink PLA assay to analyze the interaction between Cdh1 and MAST1 using specific antibodies. Scale bar: 10 µm. **(E)** Schematic representation of full length Cdh1 (1-496 aa) encoding WD40 domain (represented as Cdh1-WT), N-terminus Cdh1 (1-155 aa) lacking WD40 domain (represented as Cdh1-CTM1), and C-terminus Cdh1 (156-496 aa) encoding WD40 domain (represented as Cdh1-CTM2). Interactions between full length MAST1 and Cdh1 truncated mutants by co-immunoprecipitation and immunoblotting with the indicated antibodies (lower panel). **(F)** Schematic representation of full length MAST1 (1-1570 aa) encoding serine/threonine (S/T) kinase domain and PDZ domain (represented as MAST1-WT), N-terminus MAST1 (1-832 aa) encoding S/T kinase domain (represented as MAST1-MTM1), C-terminus MAST1 (833-1570 aa) encoding PDZ domain (represented as MAST1-MTM2), and C-terminus MAST1 (1118-1465 aa) lacking PDZ domain (represented as MAST1-MTM3). Interactions between full length Cdh1 and MAST1 truncated mutants by co-immunoprecipitation and immunoblotting with the indicated antibodies (lower panel). **(G)** The effect of Cdh1 on endogenous MAST1 protein was analyzed in HeLa cells transfected with increasing concentrations of Flag-Cdh1. **(H)** HeLa cells were transfected with sgRNA1 and sgRNA2 targeting *Cdh1* to assess the endogenous protein levels of Cdh1 and MAST1 by Western blotting. **(I)** The Cdh1-mediated degradation of endogenous MAST1 protein was rescued in cells transfected with sgRNA targeting *Cdh1*. **(J)** The ubiquitination of endogenous MAST1 was analyzed by transfecting HeLa cells with Flag-Cdh1 or sgRNA targeting *Cdh1* followed by immunoprecipitation with an anti-MAST1 antibody and immunoblotting with an anti-ubiquitin antibody. Protein expression was checked by Western blotting with the indicated antibodies. GAPDH was used as a loading control.

The dose-dependent treatment with the proteasomal inhibitor MG132 resulted in a gradual increase in endogenous MAST1 protein (Figure S4A). Furthermore, the ubiquitin smear was observed in HEK293 cells transfected with both MAST1 and the ubiquitin constructs, indicating that MAST1 undergoes 26S proteasomal degradation (Figure S4B). Therefore, we wished to identify which E3 ligases regulate MAST1 protein degradation. Among several E3 ligases, we found that Cdh1 significantly reduced MAST1 protein, along with CHIP, a previously reported E3 ligase for MAST1 12 (Figure 4A).

To explore the molecular mechanism by which Cdh1 regulates MAST1, we analyzed the physical association between them. Co-immunoprecipitation with endogenous Cdh1 or MAST1 antibodies revealed that Cdh1 co-precipitated with MAST1, and vice versa (Figure 4B). A similar interaction was observed between exogenous Cdh1 and MAST1 (Figure 4C). Additionally, the *in situ* interaction between Cdh1 and MAST1 was confirmed using a Duolink PLA assay (Figure 4D). Next, we sought to identify which region of Cdh1 interacts with MAST1, and *vice versa*. We generated two Cdh1 truncated mutants (CTMs), i.e., N-terminus Cdh1 (CTM1: 1-155 aa) lacking the WD40 domain and C-terminus Cdh1 (CTM2: 156-496 aa) encoding the WD40 domain (Figure 4E, upper panel). The co-immunoprecipitation assays showed that CTM2 interacted with full length MAST1, while CTM1 did not show any interaction with MAST1 (Figure 4E, lower panel). It is known that the WD40 domain is essential for interaction with its binding partners 38, 39, here we also observed that the WD40 domain in the C-terminus region of Cdh1 was necessary to interact with MAST1. On the other hand, MTM2 having a PDZ domain interacted with full length Cdh1, while MTM1 and MTM3 did not interact with Cdh1 (Figure 4F), suggesting that the PDZ domain of MAST1 is critical for binding with Cdh1.

An increase in Flag-Cdh1 produced a dose-dependent decrease in MAST1 protein (Figure 4G), whereas depletion of Cdh1 upregulated the MAST1 protein level (Figure 4H). The effect of Cdh1 depletion on the MAST1 protein level was reversed when Cdh1-depleted cells were reconstituted with Flag-Cdh1 (Figure 4I, lane 4 vs. 3). The effect of Cdh1 on exogenous MAST1 was cross-confirmed in HEK293 cells (Figure S5A-C).

Next, we analyzed the effect of Cdh1 on MAST1 ubiquitination status using co-immunoprecipitation assays. Overexpression of Cdh1 showed a significant increase in the polyubiquitination smear on MAST1, while sgRNA targeted *Cdh1* depletion reduced the ubiquitination smear on MAST1 protein (Figure 4J, lane 3 vs 2). The effect of Cdh1 on MAST1 ubiquitination was reversed when cells were transfected with sgRNA1 targeting *Cdh1* (Figure 4J, lane 4 vs 2), indicating that Cdh1 destabilizes MAST1 protein by promoting its ubiquitination. Furthermore, the biological significance of Cdh1 on MAST1-mediated cisplatin resistance showed that the overexpression of Cdh1 increased cytotoxicity of cisplatin in HeLa-cis^R^ cells, while knockdown of *Cdh1* reduced cytotoxicity of cisplatin in HeLa-cis^R^ cells as evidenced by increased cell viability (Figure S5D).

### USP1 deubiquitinates MAST1

**Figure 5:**
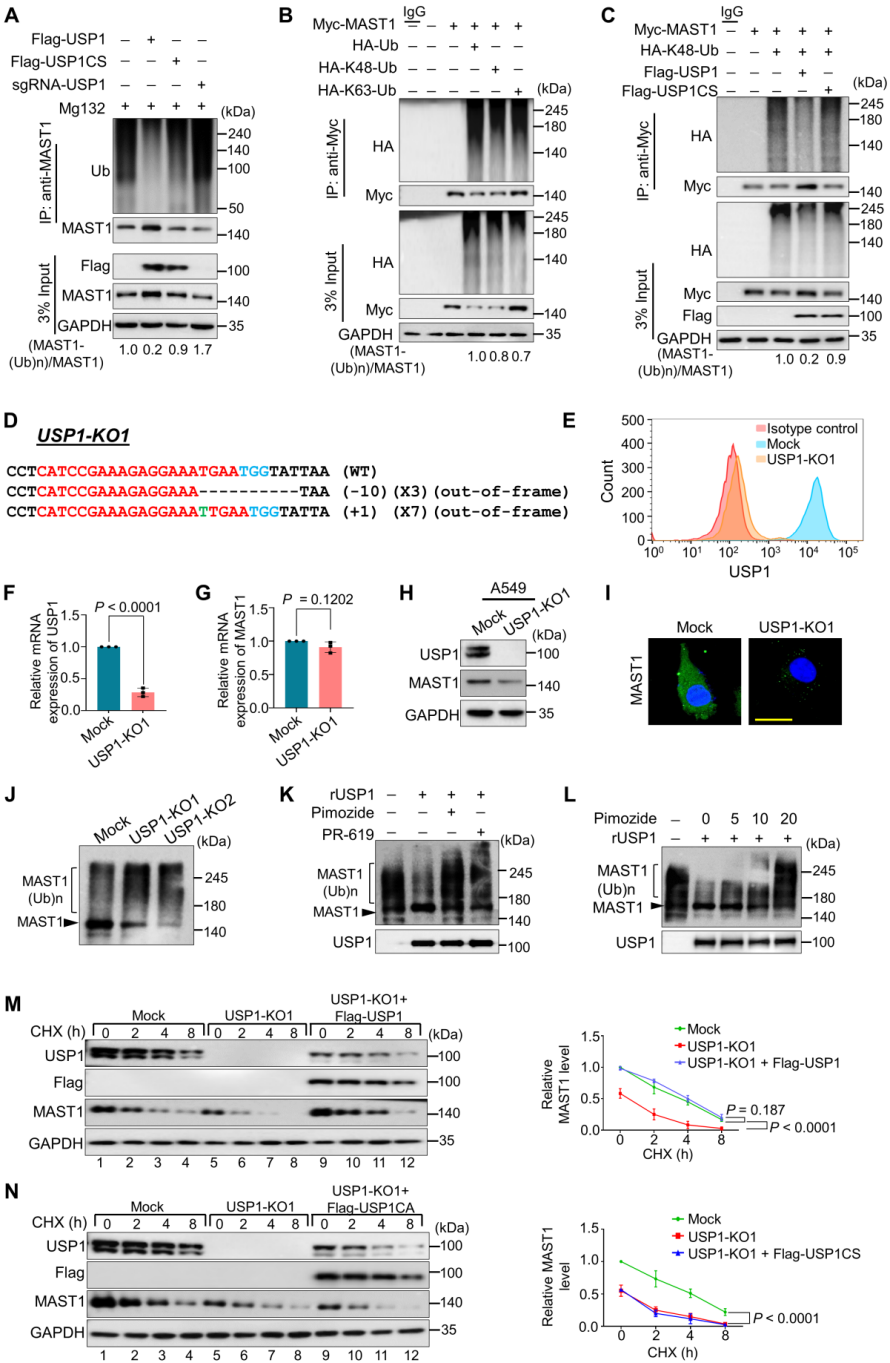
** USP1 extends MAST1 protein half-life by its deubiquitinating activity. (A)** The ubiquitination and deubiquitination of endogenous MAST1 were analyzed by transfecting HeLa cells with Flag-USP1, Flag-USP1CS, or sgRNA targeting *USP1* followed by immunoprecipitation with an anti-MAST1 antibody and immunoblotting with an anti-ubiquitin antibody. The cells were treated with MG132 for 6 h prior to harvest. **(B)** The K48- and K63-linked polyubiquitination of MAST1 was analyzed by transfecting HEK293 cells with Myc-MAST1, HA-ubiquitin, HA-K48-ubiquitin, and HA-K63-ubiquitin, followed by immunoprecipitation with an anti-Myc antibody and immunoblotting with anti-HA and anti-Myc antibodies. **(C)** The deubiquitination of K48-linked ubiquitination of MAST1 by USP1 was analyzed by transfecting HEK293 cells with Myc-MAST1 and HA-K48-ubiquitin along with Flag-USP1 or Flag-USP1CS, followed by immunoprecipitation with an anti-Myc antibody and immunoblotting with anti-HA and anti-Myc antibodies. The relative protein expression of MAST1-(Ub)n with respect to input MAST1 for (A-C) was quantified using ImageJ software and represented as (MAST1-(Ub)n/MAST1) below the blot. **(D)** Sanger sequencing data showing the disrupted *USP1* gene sequences in A549 cells (USP1-KO1). The sgRNA recognition site is denoted in red. The deleted bases are indicated with dashes, and the inserted bases are denoted with green, with the number of deleted or inserted bases indicated in parentheses. The number of occurrences of the indicated sequence is shown in parentheses (for example, X3 and X7 indicate the number of each clone sequenced).** (E)** Flow cytometry assay showing the expression of USP1 in mock control vs. USP1-KO1. **(F)** The effect of USP1-KO1 on the mRNA expression of *USP1* and **(G)** *MAST1* was analyzed by qRT-PCR with specific primers. The relative mRNA expression levels are shown after normalization to GAPDH mRNA expression. Data are presented as the mean and standard deviation of three independent experiments (n = 3). A two-tailed *t*-test was used, and the *P* values are indicated. **(H)** Western blot analysis of the endogenous expression of USP1 and MAST1 protein in USP1-KO1. GAPDH was used as the internal loading control. **(I)** The effect of *USP1* gene disruption on the endogenous expression of MAST1 was analyzed by immunofluorescence staining. Scale bar: 10 µm. **(J)** The TUBEs assay was performed to assess the ubiquitination status of the MAST1 protein in mock control and USP1-KO1 and USP1-KO2 clones. Cell lysates were immunoprecipitated with TUBEs antibodies, followed by immunoblotting with the indicated antibodies. **(K)** The total polyubiquitinated MAST1 protein was pulled down using TUBE2 resin from USP1-KO1 A549 cells treated with or without rUSP1 protein in the presence or absence of PR-619 (100 µM) and pimozide (20 µM) at 37 °C for 1 h. The eluted samples were analyzed by Western blotting with indicated antibodies. **(L)** The polyubiquitinated MAST1 protein was pulled down using TUBE2 resin treated with or without rUSP1 protein in the presence or absence of increasing concentrations of pimozide (0, 5, 10, and 20 µM) at 37 °C for 1 h. The eluted samples were analyzed by Western blotting with indicated antibodies. **(M)** Mock control, USP1-KO1, and USP1-KO1 reconstituted with either Flag-USP1 or** (N)** Flag-USP1CS were used to analyze the half-life of MAST1. CHX (150 µg/mL) was administered for the indicated time, and the cells were then harvested for Western blotting with the indicated antibodies, GAPDH was used as a loading control. Data are presented as the mean and standard deviation of three independent experiments (n=3). Two-way ANOVA followed by Tukey's post hoc test was used with the indicated *P* values.

We analyzed the deubiquitinating activity of USP1 on both endogenous and exogenous MAST1. USP1 produced a significant decrease in the polyubiquitination of both endogenous (Figure 5A, lane 2 vs. 1) and exogenous MAST1 (Figure S6A, lane 4 vs. 3). However, no deubiquitinating activity on the MAST1 protein was observed in the presence of USP1CS (Figure 5A, lane 3; Figure S6A, lane 5) or upon depletion of *USP1* by sgRNA (Figure 5A, lane 4) or shRNA (Figure S6A, lane 6), indicating that USP1 deubiquitinates MAST1 protein. We next found that overexpression of USP1 significantly reduced Cdh1-mediated MAST1 ubiquitination (Figure S6B, lane 6 vs. 5). Furthermore, we investigated the type of polyubiquitin chains with which MAST1 protein can interact. The co-immunoprecipitation assays showed both Lys(K)-48- and Lys(K)-63-linked polyubiquitination smear on MAST1 protein (Figure 5B). Thus, we investigated whether USP1 deubiquitinates K48- or K63-linked polyubiquitination of MAST1. Our results showed that USP1 can deubiquitinate only K48- (Figure 5C) but not K63-linked polyubiquitination of MAST1 (Figure S7), suggesting that USP1 regulates MAST1 proteolysis through the proteasomal pathway.

### Loss of USP1 promotes MAST1 protein degradation

We applied the CRISPR/Cas9 system to generate single-cell-derived *USP1* knockout clones in A549 cells, which is a well-established cell line for studying cisplatin-resistance mechanisms. The highly efficient sgRNA targeting exon 5 of *USP1* was co-transfected with Cas9 and subjected to single-cell clonal selection. Disruption of the* USP1* gene showed cleavage by the T7E1 assay (Figure S8A) and reduction in USP1 protein level by Western blotting (Figure S8B). The T7E1-positive *USP1* knockout clone #14 (hereafter USP1-KO1) and *USP1* knockout clone #23 (hereafter USP1-KO2) displayed out-of-frame mutations (Figure 5D and Figure S9A) and significant reduction in USP1 expression by flow cytometry compared with the mock control (Figure 5E). The RT-PCR analysis showed significant reduction in the mRNA levels of *USP1* in USP1-KO1 and USP1-KO2 clones compared with the mock control (Figure 5F and Figure S9B). However, *USP1* depletion did not produce any significant changes in the *MAST1* mRNA level (Figure 5G and Figure S9C), indicating that USP1 does not transcriptionally regulate MAST1 expression. We further validated the effect of USP1 depletion on MAST1 protein expression in USP1KO clones. The loss of *USP1* significantly decreased the MAST1 protein level, as shown by Western blotting (Figure 5H and Figure S9D) and immunostaining (Figure 5I), compared with the respective mock controls. Moreover, the off-target analysis of sgRNA targeting *USP1* showed no non-specific cleavages in the USP1KO clone (Figure S10).

To determine the effect of USP1 on the polyubiquitination status of the MAST1 protein, we utilized ubiquitin-binding TUBE2 resin to purify polyubiquitinated MAST1 from cellular extracts of USP1KO clones and performed TUBEs assay 40. We observed a higher ubiquitin-conjugated smear on the MAST1 protein in both USP1-KO1 and USP1-KO2 clones compared with the mock control (Figure 5J), suggesting that loss of USP1 signals MAST1 for rapid protein degradation. We next analyzed whether recombinant USP1 (rUSP1) could remove polyubiquitin chains conjugated to MAST1 protein. To this end, we performed *in vitro* deubiquitination assay on polyubiquitinated MAST1 by treating with rUSP1. The USP1-KO1 cells showing higher ubiquitin-conjugated smear on MAST1 was significantly reduced when USP1-KO1 samples were incubated with rUSP1, while the activity of rUSP1 was drastically inhibited in the presence of DUB inhibitor (PR-619) or USP1 inhibitor (pimozide) (Figure 5K). We further assessed the effect of dose-dependent increase of pimozide on USP1 activity. The results showed a gradual reduction in deubiquitinating activity of rUSP1 on MAST1 as evidenced by increased polyubiquitination smear on MAST1 (Figure 5L).

### USP1 extends the half-life of MAST1

To demonstrate the half-life of the MAST1 protein, we treated cells with cycloheximide (CHX) for 0 to 9 h. The half-life of MAST1 was estimated to be around 4 h (Figure S11A). However, the half-life of MAST1 was drastically reduced in the presence of Cdh1, an E3 ligase for MAST1 protein (Figure S11B), suggesting that Cdh1 regulates MAST1 protein turnover. To corroborate the role of USP1 on MAST1 protein turnover, we analyzed the half-life of MAST1 in the presence and absence of USP1. USP1 depletion shortened the half-life of MAST1 (Figure 5M, lanes 5-8), which was rescued when the USP1-depleted cells were reconstituted with Flag-USP1 (Figure 5M, lanes 9-12), while Flag-USP1CS failed to rescue the half-life (Figure 5N, lanes 9-12). These results indicate that USP1 regulates MAST1 protein turnover and extends its half-life.

### Correlation between USP1 and MAST1 expression across a wide range of cancer types

**Figure 6:**
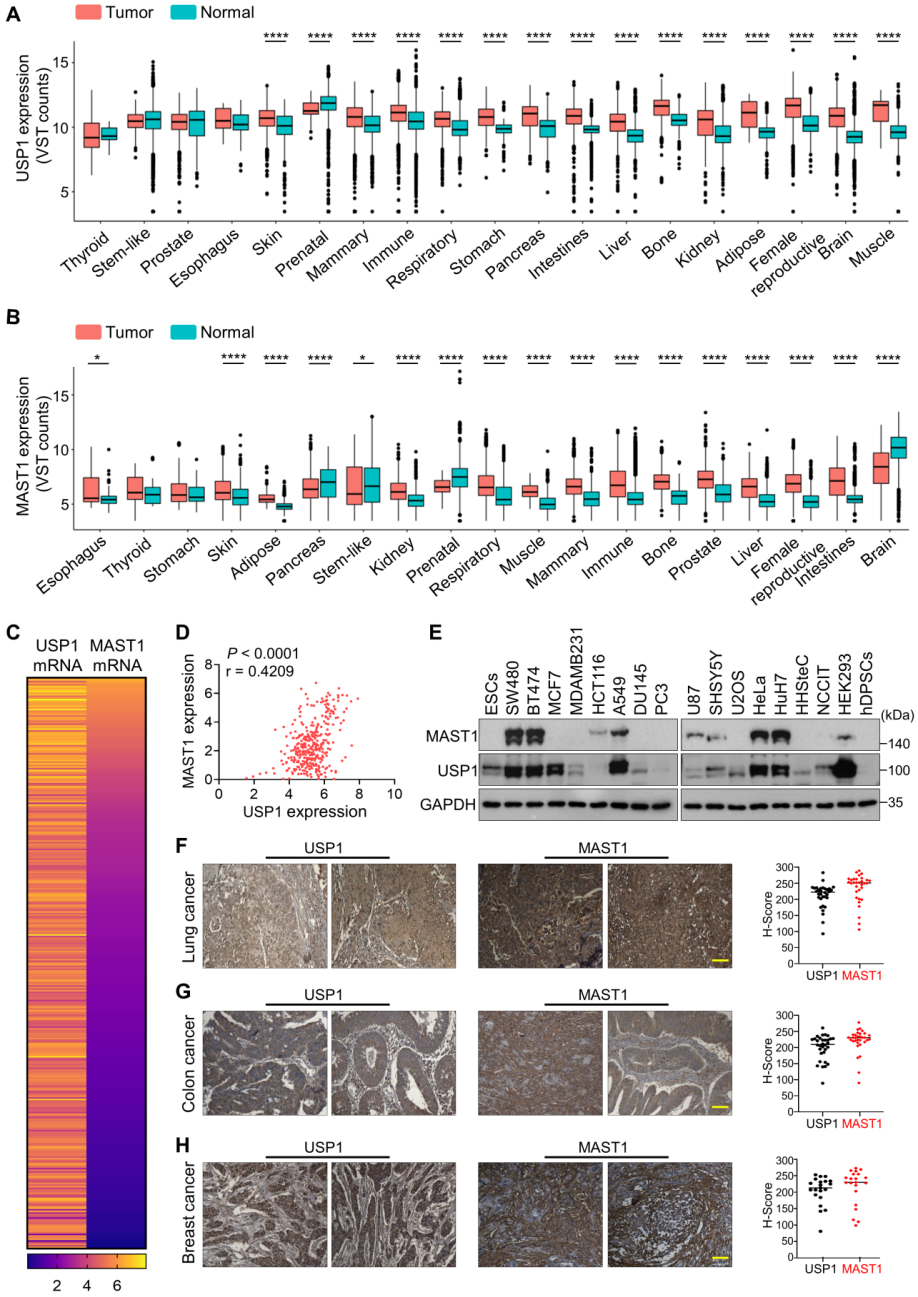
**Clinical correlation between USP1 and MAST1 expression in various cancer tissues. (A)** Box plot showing the difference between USP1 expression in tumor and normal tissues using Correlation AnalyzeR. Significance was determined via the Wilcoxon rank sum test: ****P < 0.0001. **(B)** Box plot showing the difference between MAST1 expression in tumor and normal tissues using Correlation AnalyzeR. Significance was determined via the Wilcoxon rank sum test: *P < .05, ****P < 0.0001. VST stands for variance-stabilizing transform. **(C)** A heat map showing mRNA expression levels of USP1 and MAST1 derived from the CCLE database. Representative samples are arranged from high to low mRNA levels of MAST1, and corresponding USP1 values are sorted. **(D)** A scatterplot showing the expression correlation between USP1 and MAST1 mRNA levels. Pearson correlations (r) quantifying the relationship between USP1 and MAST1 are given. **(E)** Endogenous protein expression patterns of USP1 and MAST1 in different cancer and non-cancer cell lines were assessed by Western blotting. GAPDH was used as the loading control.** (F-H)** Representative immunohistochemical (IHC) staining images of endogenous USP1 and MAST1 in (F) human lung cancer (n = 32), (G) colon cancer (n = 32), and (H) breast cancer (n = 21) tissues. All IHC images were quantified with an H-score. Scale bar = 30 µm.

We used Correlation AnalyzeR to evaluate the expression of *USP1* and *MAST1* in different types of cancer tissues 41. We found that both *USP1* (Figure 6A, Figure S12) and *MAST1* (Figure 6B, Figure S13) are more highly expressed in cancer than in normal tissue, and we confirmed this observation by immunohistochemistry using human tissue samples (Figure S14). Considering the high expression of both USP1 and MAST1 in cancer tissues, we next used the Cancer Cell Line Encyclopedia (CCLE) database to analyze *USP1-MAST1* correlation at mRNA level. The high scores of *MAST1* mRNA corresponded with *USP1* mRNA level across a wide range of cancer types (Figure 6C, Table S6). Moreover, a scatterplot of the *USP1-MAST1* expression patterns with an r value of 0.4209 across different tissues suggested the existence of a positive correlation between USP1 and MAST1 (Figure 6D).

We further validated the correlation between USP1 and MAST1 protein in several cancer cell lines by Western blotting. The high expression of USP1 corresponded with MAST1 protein expression in the cancer cell lines we tested (Figure 6E). To determine the clinical relevance of our finding, we performed immunohistochemistry to analyze USP1 and MAST1 expression in human clinical tissue samples. We observed significantly high expression patterns for both USP1 and MAST1 in lung cancer (n = 32), colon cancer (n = 32), and breast cancer (n = 21) samples (Figure 6F-H, Figure S15). Collectively, these results clinically validate our findings and support the positive correlation of USP1 and MAST1 across different cancer types.

### Loss of USP1 induces DNA damage and apoptosis

**Figure 7:**
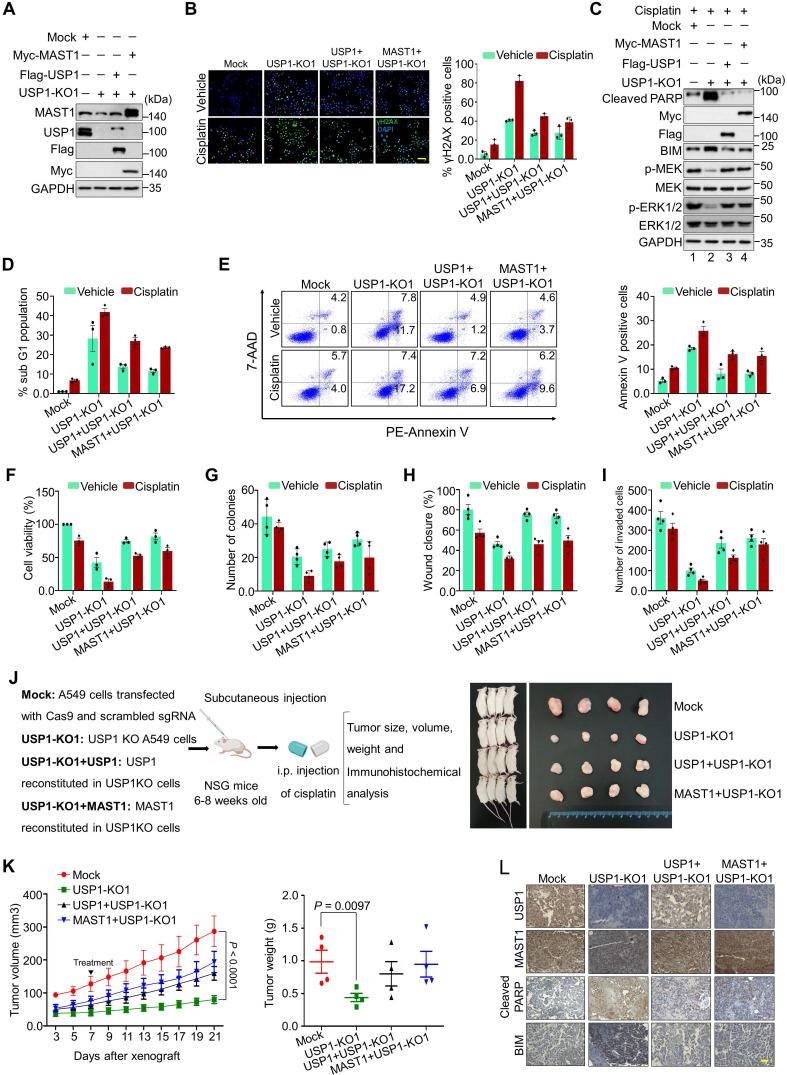
** Depletion of USP1 promotes apoptosis, DNA damage, and tumor growth arrest.** Mock control, USP1-KO1, and USP1-KO1 cells reconstituted with either USP1 or MAST1 were used to perform the following experiments. **(A)** Western blot analyses to validate the expression of USP1 and MAST1 using USP1- and MAST1-specific antibodies. GAPDH was used as the loading control.** (B)** The cells were treated with either vehicle or cisplatin (2 µg/mL) for 24 h and subjected to immunofluorescence analysis to estimate γH2AX foci formation. Green, γH2AX; blue, nucleus stained by DAPI. Scale bar = 100 µm. The right panel depicts the percentage of γH2AX-positive cells. **(C)** The cells were treated with cisplatin (2 µg/mL) for 24 h, and MEK1 activation and apoptosis-related factors were determined using Western blotting. GAPDH was used as the internal loading control. **(D)** The cells were treated with either vehicle or cisplatin (2 µg/mL) for 48 h and subjected to flow cytometry to measure the DNA content using PI staining and **(E)** annexin-V and 7-AAD staining. **(F)** The cells were treated with a sub-lethal dose of cisplatin (2 µg/mL) for 48 h, and cell viability was assayed using CCK-8 reagent. Data are presented as the mean and standard deviation of three independent experiments (n = 3). **(G-I)** Vehicle- or cisplatin-treated cells were subjected to a **(G)** colony formation assay, **(H)** wound-healing assay, and **(I)** Transwell cell-invasion assay. Data are presented as the mean and standard deviation of four independent experiments (n = 4). **(J)** Xenografts were generated by subcutaneously injecting the mentioned cell groups into the right flanks of NSG mice (n = 4/group). Mice were i.p. injected with either saline (vehicle) or cisplatin (2 mg/kg) twice a week beginning 7 days after xenograft implantation, and tumor size was monitored. Tumor volumes were recorded, and tissues were stored for IHC experiments. The right panel shows the tumors excised from the mice after the experiment. **(K)** Tumor volume was measured every other day and is presented graphically. Data are presented as the mean and standard deviation of four independent experiments (n = 4). Two-way ANOVA followed by Tukey's post hoc test was used with the indicated *P* values.** (L)** Xenograft tumors were embedded in paraffin and sectioned. IHC analyses were performed with the indicated antibodies. Scale bar = 30 µm.

We used USP1-KO1 and USP1-KO2 clones having low MAST1 protein level, and these USP1KO clones reconstituted with either USP1 or MAST1 expressing high MAST1 protein (Figure 7A and Figure S16A), to explore the molecular mechanisms of USP1 regulation on MAST1-driven cisplatin-resistance. These USP1KO clones were subjected to further functional studies. We first checked the extent of DNA damage induced by cisplatin in USP1KO clones by estimating γH2AX expression. Both USP1-KO1 and USP1-KO2 clones showed greater γH2AX foci formation than the mock control (Figure 7B and Figure S16B), suggesting that loss of USP1 leads to cisplatin-mediated DNA damage. In contrast, reconstitution with USP1 or MAST1 reduced γH2AX foci formation (Figure 7B and Figure S16B). We further analyzed the MAST1-mediated MEK1 activation and its downstream ERK1/2 phosphorylation in cisplatin-treated USP1-depleted cells. Interestingly, cisplatin-treated USP1-KO1 cells showed decreased expression of phosphorylated MEK1 and ERK (Figure 7C, lane 2). The reduced phosphorylation of MEK1 and ERK was restored by reconstitution with USP1 or MAST1 (Figure 7C, lanes 3 and 4). Moreover, our evaluation of apoptotic factors revealed that BIM and cleaved PARP showed elevated expression in cisplatin-treated USP1KO cells (Figure 7C, lane 2). USP1-depleted cells also showed an increase in their sub-G1 populations (Figure 7D) and annexin-V-positive cells (Figure 7E), whereas cisplatin was considerably less toxic in USP1-depleted cells reconstituted with USP1 or MAST1 (Figure 7D, E).

### USP1 regulates MAST1-mediated tumor progression

To analyze the influence of USP1 on MAST1-mediated development of cisplatin-resistance during tumor progression, we performed several assays related to carcinogenesis. Cisplatin-treated USP1-KO1 and USP1-KO2 clones showed lower cell viability, and this decreased cell viability was rescued by reconstitution with USP1 or MAST1 (Figure 7F and Figure S16C). An anchorage-independent colony formation assay showed reduced colony numbers when USP1-KO1 (Figure 7G and Figure S17A), and USP1-KO2 cells were treated with cisplatin compared with cisplatin-treated mock control (Figure S18A). Likewise, the cellular migration and invasion of cisplatin-treated USP1-KO1 (Figure 7H, I and Figure S17B,C) and USP1-KO2 cells were significantly hampered compared with the mock control (Figure S18B, C). However, the cisplatin toxicity in USP1-KO1 and USP1-KO2 cells was reduced by reconstitution with USP1 or MAST1, as evidenced by increased colony numbers, cell migration and invasion (Figure 7G-I, Figure S17 and S18).

Next, to validate our findings, we performed *in vivo* studies by subcutaneously injecting the cells from Figure 7A into the right flanks of NOD scid γ (NSG) mice. Cisplatin-treated mice bearing USP1KO tumors displayed a significant reduction in tumor volume and weight, compared with the cisplatin-treated mock group. However, reconstitution with either USP1 or MAST1 resulted in significant increase in tumor volume and weight (Figure 7J, K). Furthermore, the immunohistochemical analysis of the xenograft tumor tissues showed significantly reduced USP1 and MAST1 expression in the USP1KO tumors compared with the mock xenograft, and that expression was regained by reconstitution with USP1 or MAST1 (Figure 7L). Additionally, increased apoptotic markers such as BIM and cleaved PARP were observed in the USP1KO xenografts (Figure 7L). Altogether, our results showed that, upon cisplatin treatment, USP1-depletion downregulates MAST1 level and induces apoptosis that hampers tumor progression.

### Combined inhibition of USP1 and MAST1 further reduces MAST1 level and sensitizes cancer cells to cisplatin

**Figure 8:**
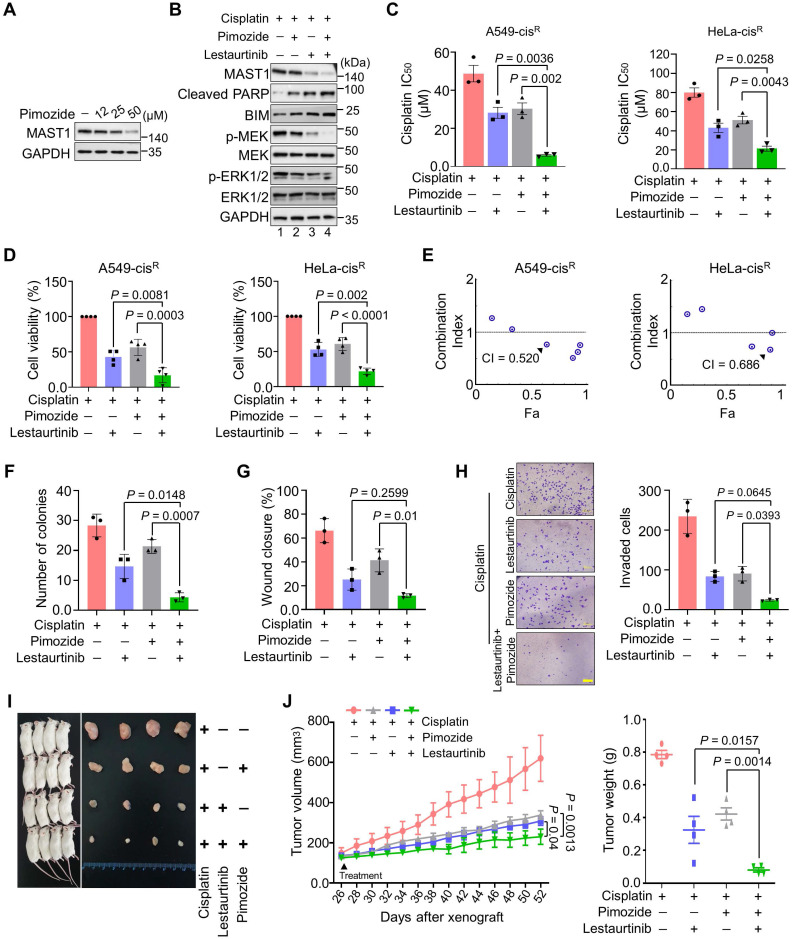
** Combination of pimozide and lestaurtinib inhibits MAST1 protein and cisplatin-resistant tumor growth more than either single treatment. (A)** The effect of USP1 inhibition on MAST1 protein level was determined by treating HeLa-cis^R^ cells with increasing concentrations of pimozide for 24 h. The protein expression of MAST1 was determined by Western blotting. GAPDH was used as an internal loading control. **(B)** The effect of combination treatment of pimozide and lestaurtinib on MAST1-mediated MEK phosphorylation. HeLa-cis^R^ cells were treated with pimozide (50 µM) and lestaurtinib (200 nM) in the presence of sub-lethal doses of cisplatin (5 µg/mL) for 24 h. The activity of MAST1 was assessed by a Western blot analysis of the phospho-MEK1 and phospho-ERK levels. GAPDH was used as an internal loading control. **(C, D)** The effect of combined treatment with pimozide and lestaurtinib on **(C)** cisplatin sensitivity (n = 3) **(D)** and cell viability in A549-cis^R^ and HeLa-cis^R^ cells (n = 4). Data are presented as the mean and standard deviation of at least three independent experiments. Two-way ANOVA followed by Tukey's post hoc test was used with the indicated *P* values. **(E)** Combination index (CI) plots for the synergistic effect of pimozide and lestaurtinib in A549-cis^R^ and HeLa-cis^R^ cells. **(F-H)** The effect of combination treatment with pimozide and lestaurtinib was validated using **(F)** colony formation assay, **(G)** wound-healing assay, and **(H)** Transwell cell-invasion assay. Data are presented as the mean and standard deviation of three independent experiments (n = 3). Two-way ANOVA followed by Tukey's post hoc test was used with the indicated *P* values. **(I)** Xenografts were generated by subcutaneously injecting A549-cis^R^ cells into the right flanks of NSG mice (n = 4). Mice were treated with pimozide (10 mg/kg), lestaurtinib (20 mg/kg), and cisplatin (5 mg/kg) beginning 26 days after xenograft implantation, and tumor size was monitored. The right panel shows the tumors excised from the mice after the experiment. **(J)** Tumor volume and tumor weight were measured and are presented graphically. Data are presented as the mean and standard deviation of four independent experiments (n = 4). Two-way ANOVA followed by Tukey's post hoc test was used with the indicated *P* values. For brevity, statistical significance is shown only for comparisons between the groups of interest, except for the negative control group.

Our findings suggest that USP1 depletion destabilizes MAST1, which facilitates cisplatin sensitization in cancer. Therefore, we first checked the effect of pimozide, a previously identified USP1 inhibitor 19, in cisplatin-resistant HeLa cells. Upon exposure to increasing concentrations of pimozide, MAST1 protein level gradually diminished (Figure 8A). Thus, we hypothesized that combined pharmacological inhibition of USP1 and MAST1 might improve the effects of MAST1-based therapy in cisplatin-resistant tumors. To this end, we applied pimozide and lestaurtinib (a MAST1 inhibitor) 8 and examined their combined effects in sensitizing cisplatin-resistant tumors. The pimozide and lestaurtinib combination treatment significantly reduced MAST1 expression and the subsequent phosphorylation of MEK1 and ERK compared with the individual treatments (Figure 8B, lane 4).

The combination of pimozide and lestaurtinib synergistically sensitized cells to cisplatin (Figure 8C) and further reduced the viability of cisplatin-resistant A549 and HeLa cells (Figure 8D), with a combination index (CI) of 0.520-0.686 (Figure 8E). Pimozide and lestaurtinib together also significantly hampered cell growth (Figure S19A) and *in vitro* tumorigenicity, as displayed in colony formation (Figure 8F, Figure S19B), wound healing (Figure 8G, Figure S19C), and cell invasion (Figure 8H) assays.

Lastly, we examined the therapeutic efficiency of a single treatment of pimozide, lestaurtinib, and their combination in NSG mouse xenografts. The combined targeting of USP1 and MAST1 by pimozide and lestaurtinib synergistically enhanced cisplatin sensitivity in the mouse xenografts, significantly reducing tumor volume and weight compared with a single treatment of pimozide or lestaurtinib (Figure 8I, J). Overall, our results suggest that the pharmacological inhibition of both MAST1 and USP1 could mitigate cisplatin-resistance in cancerous tumors.

## Discussion

A large proportion of anticancer drugs are DNA crosslinkers that inflict DNA damage and cause apoptosis in cancer cells. Cisplatin is a platinum-based drug successfully used to treat several human cancers; however, tumor recurrence due to the acquisition of cisplatin-resistance is a major therapeutic limitation. Several factors are directly or indirectly involved in driving cisplatin-resistance in human cancers 4. Recently, Jin *et al*. identified MAST1 as an essential driver of cisplatin-resistance in tumors by activation of the MEK pathway in a cRaf-independent manner 8. Later, the same group identified chaperone hsp90B as a binding partner and protein stabilizer of MAST1 in cancers. The inhibition of hsp90 promotes E3 ligase CHIP-mediated ubiquitination of MAST1, resulting in cisplatin sensitization of cancer cells 12 and suggesting that MAST1 protein level is a key factor in circumventing cisplatin-resistance. Elevated MAST1 expression in cancers is one factor hampering the therapeutic success of cisplatin-based treatment. Therefore, identifying the protein stabilizers of MAST1 is a top priority.

Since deubiquitinases stabilize their substrates by counteracting their protein degradation, we initiated this study to systematically perform genome-wide screening for DUBs that regulate MAST1 protein levels. In parallel, we also screened for DUBs that confer cisplatin-resistance in cancers. Our dual screening approach based on a CRISPR/Cas9 system identified USP1 as a novel and bona fide candidate that governs cisplatin-resistance in cancer. USP1 has previously been implicated in the DNA damage response through its regulation of the monoubiquitination of PCNA and FANCD2/I 33, 42, 43. Here, we demonstrated that USP1 interacts with, deubiquitinates, and stabilizes MAST1 expression by regulating its protein turnover. We provide evidence that USP1 mediates MAST1 stability by preventing Cdh1-linked MAST1 protein degradation. We further delineate the mechanism by which the depletion of USP1 downregulates MAST1-mediated activation of MEK1 and its downstream ERK1/2 in cancers.

Cisplatin treatment leads to replication fork damage, which results in high γH2AX expression, an indicator of cisplatin-mediated cell death 32. We demonstrated that the loss of USP1 promoted γH2AX expression, induced pro-apoptotic factors, and subsequently increased cancer sensitivity to cisplatin treatment both *in vitro* and *in vivo*. Therefore, USP1 could be therapeutically targeted along with MAST1 to synergistically improve the value of cisplatin-based therapeutics in cisplatin-resistant tumors. Previously, USP1-depletion has been associated with increased cisplatin sensitivity in cancers 15, 18, 44. Recently, a USP1 inhibitor was adapted to sensitize human cisplatin-resistant NSCLC cells 19, and it also destabilizes Snail expression and increases platinum sensitivity in ovarian cancer 15. In this study, pharmacological inhibition of USP1 and MAST1 using pimozide and lestaurtinib synergistically boosted the effect of cisplatin toxicity on tumor growth. Deciphering the molecular mechanism by which the ubiquitin proteasomal system regulates MAST1 protein turnover might be an effective way to advance MAST1-based therapy for cancer patients and overcome cisplatin-resistance.

## Supplementary Material

Supplementary figures and tables.

## Abbreviations

CCLE: Cancer Cell Line Encyclopedia
DUBs: Deubiquitinating enzymes
ERK: Extracellular signal-regulated kinase 1/2
MAPK: Mitogen-activated protein kinase
MAST1: Microtubule-associated serine/threonine-protein kinase 1
NSG: NOD scid γ
sgRNA: Single guide RNA
shRNA: Short hairpin RNA
USPs: Ubiquitin-specific proteases

## References

[1] Galanski M (2006). Recent developments in the field of anticancer platinum complexes. Recent patents on anti-cancer drug discovery.

[2] Kang X, Xiao HH, Song HQ, Jing XB, Yan LS, Qi RG (2015). Advances in drug delivery system for platinum agents based combination therapy. Cancer biology & medicine.

[3] Siddik ZH (2003). Cisplatin: mode of cytotoxic action and molecular basis of resistance. Oncogene.

[4] Galluzzi L, Senovilla L, Vitale I, Michels J, Martins I, Kepp O (2012). Molecular mechanisms of cisplatin resistance. Oncogene.

[5] Wang J, Zhou JY, Wu GS (2007). ERK-dependent MKP-1-mediated cisplatin resistance in human ovarian cancer cells. Cancer research.

[6] Wang C, Zhou Z, Subhramanyam CS, Cao Q, Heng ZSL, Liu W (2020). SRPK1 acetylation modulates alternative splicing to regulate cisplatin resistance in breast cancer cells. Communications biology.

[7] Achkar IW, Abdulrahman N, Al-Sulaiti H, Joseph JM, Uddin S, Mraiche F (2018). Cisplatin based therapy: the role of the mitogen activated protein kinase signaling pathway. Journal of translational medicine.

[8] Jin L, Chun J, Pan C, Li D, Lin R, Alesi GN (2018). MAST1 Drives Cisplatin Resistance in Human Cancers by Rewiring cRaf-Independent MEK Activation. Cancer cell.

[9] Garland P, Quraishe S, French P, O'Connor V (2008). Expression of the MAST family of serine/threonine kinases. Brain research.

[10] Lumeng C, Phelps S, Crawford GE, Walden PD, Barald K, Chamberlain JS (1999). Interactions between beta 2-syntrophin and a family of microtubule-associated serine/threonine kinases. Nature neuroscience.

[11] Robinson DR, Kalyana-Sundaram S, Wu YM, Shankar S, Cao X, Ateeq B (2011). Functionally recurrent rearrangements of the MAST kinase and Notch gene families in breast cancer. Nature medicine.

[12] Pan C, Chun J, Li D, Boese AC, Li J, Kang J (2019). Hsp90B enhances MAST1-mediated cisplatin resistance by protecting MAST1 from proteosomal degradation. The Journal of clinical investigation.

[13] Pan C, Kang J, Hwang JS, Li J, Boese AC, Wang X (2021). Cisplatin-mediated activation of glucocorticoid receptor induces platinum resistance via MAST1. Nature Communications.

[14] Tanguturi P, Kim KS, Ramakrishna S (2020). The role of deubiquitinating enzymes in cancer drug resistance. Cancer chemotherapy and pharmacology.

[15] Sonego M, Pellarin I, Costa A, Vinciguerra GLR, Coan M, Kraut A (2019). USP1 links platinum resistance to cancer cell dissemination by regulating Snail stability. Science advances.

[16] Wang SA, Young MJ, Wang YC, Chen SH, Liu CY, Lo YA (2021). USP24 promotes drug resistance during cancer therapy. Cell death and differentiation.

[17] Antao AM, Tyagi A, Kim KS, Ramakrishna S (2020). Advances in Deubiquitinating Enzyme Inhibition and Applications in Cancer Therapeutics. Cancers.

[18] Xu X, Li S, Cui X, Han K, Wang J, Hou X (2019). Inhibition of Ubiquitin Specific Protease 1 Sensitizes Colorectal Cancer Cells to DNA-Damaging Chemotherapeutics. Frontiers in oncology.

[19] Chen J, Dexheimer TS, Ai Y, Liang Q, Villamil MA, Inglese J (2011). Selective and cell-active inhibitors of the USP1/ UAF1 deubiquitinase complex reverse cisplatin resistance in non-small cell lung cancer cells. Chemistry & biology.

[20] Subramaniam D, Angulo P, Ponnurangam S, Dandawate P, Ramamoorthy P, Srinivasan P (2020). Suppressing STAT5 signaling affects osteosarcoma growth and stemness. Cell death & disease.

[21] Das S, Chandrasekaran AP, Suresh B, Haq S, Kang J-H, Lee S-J (2020). Genome-scale screening of deubiquitinase subfamily identifies USP3 as a stabilizer of Cdc25A regulating cell cycle in cancer. Cell Death & Differentiation.

[22] Chandrasekaran AP, Kaushal K, Park C-H, Kim K-S, Ramakrishna S (2021). USP32 confers cancer cell resistance to YM155 via promoting ER-associated degradation of solute carrier protein SLC35F2. Theranostics.

[23] Haq S, Sarodaya N, Karapurkar JK, Suresh B, Jo JK, Singh V (2022). CYLD destabilizes NoxO1 protein by promoting ubiquitination and regulates prostate cancer progression. Cancer Letters.

[24] Liang XJ, Shen DW, Garfield S, Gottesman MM (2003). Mislocalization of membrane proteins associated with multidrug resistance in cisplatin-resistant cancer cell lines. Cancer research.

[25] Shen D, Pastan I, Gottesman MM (1998). Cross-resistance to methotrexate and metals in human cisplatin-resistant cell lines results from a pleiotropic defect in accumulation of these compounds associated with reduced plasma membrane binding proteins. Cancer research.

[26] Ramakrishna S, Kwaku Dad AB, Beloor J, Gopalappa R, Lee SK, Kim H (2014). Gene disruption by cell-penetrating peptide-mediated delivery of Cas9 protein and guide RNA. Genome research.

[27] Nguyen TV, Li J, Lu CJ, Mamrosh JL, Lu G, Cathers BE (2017). p97/VCP promotes degradation of CRBN substrate glutamine synthetase and neosubstrates. Proceedings of the National Academy of Sciences of the United States of America.

[28] Nguyen TV (2021). USP15 antagonizes CRL4(CRBN)-mediated ubiquitylation of glutamine synthetase and neosubstrates. Proceedings of the National Academy of Sciences of the United States of America.

[29] Nguyen TV, Lee JE, Sweredoski MJ, Yang SJ, Jeon SJ, Harrison JS (2016). Glutamine Triggers Acetylation-Dependent Degradation of Glutamine Synthetase via the Thalidomide Receptor Cereblon. Molecular cell.

[30] Chou TC (2006). Theoretical basis, experimental design, and computerized simulation of synergism and antagonism in drug combination studies. Pharmacological reviews.

[31] Zhang N, Fu JN, Chou TC (2016). Synergistic combination of microtubule targeting anticancer fludelone with cytoprotective panaxytriol derived from panax ginseng against MX-1 cells *in vitro*: experimental design and data analysis using the combination index method. American journal of cancer research.

[32] Olive PL, Banáth JP (2009). Kinetics of H2AX phosphorylation after exposure to cisplatin. Cytometry Part B, Clinical cytometry.

[33] Cohn MA, Kowal P, Yang K, Haas W, Huang TT, Gygi SP (2007). A UAF1-containing multisubunit protein complex regulates the Fanconi anemia pathway. Molecular cell.

[34] Lee HJ, Zheng JJ (2010). PDZ domains and their binding partners: structure, specificity, and modification. Cell communication and signaling: CCS.

[35] Kalyoncu S, Keskin O, Gursoy A (2010). Interaction prediction and classification of PDZ domains. BMC bioinformatics.

[36] Valiente M, Andrés-Pons A, Gomar B, Torres J, Gil A, Tapparel C (2005). Binding of PTEN to specific PDZ domains contributes to PTEN protein stability and phosphorylation by microtubule-associated serine/threonine kinases. The Journal of biological chemistry.

[37] An SWA, Choi ES, Hwang W, Son HG, Yang JS, Seo K (2019). KIN-4/MAST kinase promotes PTEN-mediated longevity of Caenorhabditis elegans via binding through a PDZ domain. Aging cell.

[38] Lv XB, Xie F, Hu K, Wu Y, Cao LL, Han X (2010). Damaged DNA-binding protein 1 (DDB1) interacts with Cdh1 and modulates the function of APC/CCdh1. The Journal of biological chemistry.

[39] Meghini F, Martins T, Tait X, Fujimitsu K, Yamano H, Glover DM (2016). Targeting of Fzr/Cdh1 for timely activation of the APC/C at the centrosome during mitotic exit. Nat Commun.

[40] Hjerpe R, Aillet F, Lopitz-Otsoa F, Lang V, England P, Rodriguez MS (2009). Efficient protection and isolation of ubiquitylated proteins using tandem ubiquitin-binding entities. EMBO reports.

[41] Miller HE, Bishop AJR (2021). Correlation AnalyzeR: functional predictions from gene co-expression correlations. BMC bioinformatics.

[42] Oestergaard VH, Langevin F, Kuiken HJ, Pace P, Niedzwiedz W, Simpson LJ (2007). Deubiquitination of FANCD2 is required for DNA crosslink repair. Molecular cell.

[43] Kim JM, Parmar K, Huang M, Weinstock DM, Ruit CA, Kutok JL (2009). Inactivation of murine Usp1 results in genomic instability and a Fanconi anemia phenotype. Developmental cell.

[44] García-Santisteban I, Peters GJ, Giovannetti E, Rodríguez JA (2013). USP1 deubiquitinase: cellular functions, regulatory mechanisms and emerging potential as target in cancer therapy. Molecular Cancer.

