# Supplementary material for: CRISPR/Cas9-based genome-wide screening for deubiquitinase subfamily identifies USP1 regulating MAST1-driven cisplatin-resistance in cancer cells
Source: Theranostics. 2022 Aug 8;12(13):5949–70. doi: 10.7150/thno.72826 (PMC9373805; doi:10.7150/thno.72826)
Supplement: Supplementary file 1 — Supplementary figures and tables. [file thnov12p5949s1.pdf]

Supplementary Information

CRISPR/Cas9-based genome-wide screening for deubiquitinase subfamily identifies USP1 regulating MAST1-driven cisplatin-resistance in cancer cells

Apoorvi Tyagi[1†], Kamini Kaushal[1†], Arun Pandian Chandrasekaran[1], Neha Sarodaya[1], Soumyadip Das[1], Chang-Hwan Park[1,3], Seok-Ho Hong[2], Kye-Seong Kim[1,3]* and Suresh Ramakrishna[1,3]*

[1]Graduate School of Biomedical Science and Engineering, Hanyang University, Seoul, South Korea

[2]Department of Internal Medicine, School of Medicine, Kangwon National University, Chuncheon, South Korea

[3]College of Medicine, Hanyang University, Seoul, South Korea

† These authors contributed equally: Apoorvi Tyagi and Kamini Kaushal.

Running title: *Loss of USP1 reverses MAST1-mediated cisplatin-resistance.*

***Corresponding authors**

SR (E-mail: suri28@hanyang.ac.kr, suresh.ramakris@gmail.com)

KS (E-mail: ks66kim@hanyang.ac.kr)

Supplementary Figures

Figure S1. The efficiencies of sgRNAs and shRNAs targeting *USP1*.

Figure S2. The effect of *UAF1* depletion on MAST1 protein level and cisplatin resistance.

Figure S3. USP1 increases exogenous MAST1 protein level.

Figure S4. MAST1 protein undergoes 26S proteasomal degradation.

Figure S5. The effect of Cdh1 on MAST1 protein level and cisplatin resistance.

Figure S6. USP1 deubiquitinates Cdh1-mediated MAST1 ubiquitination.

Figure S7. The deubiquitination of K63-linked ubiquitination of MAST1 by USP1.

Figure S8. Generation of single cell-derived *USP1* knockout clones in A549 cells.

Figure S9. Validation of single cell-derived USP1KO2 clone in A549 cells.

Figure S10. Off-target analysis of sgRNA1-targeting *USP1* in USP1 knockout clone.

Figure S11. Half-life of MAST1 protein.

Figure S12. Expression of USP1 in normal vs. cancer tissues.

Figure S13. Expression of MAST1 in normal vs. cancer tissues.

Figure S14. Immunohistochemical staining of USP1 and MAST1 in human clinical samples.

Figure S15. Immunohistochemical staining of USP1 and MAST1.

Figure S16. Depletion of USP1 promotes DNA damage and prevents cell proliferation.

Figure S17. Determination of carcinogenic activity of USP1-KO1 in A549 cells.

Figure S18. Determination of carcinogenic activity of USP1-KO2 in A549 cells.

Figure S19. Combined pharmacologic inhibition of USP1 and MAST1 further sensitizes cisplatin-resistant cells to cisplatin.

Supplementary Tables

Table S1. Target sequences used for sgRNA plasmid construction.

Table S2. Target sequences used for shRNA plasmid construction.

Table S3. Oligonucleotide sequences used to get PCR amplicon for T7E1 assay.

Table S4. Oligonucleotide sequences used to get PCR amplicon for Off-target genes.

Table S5. PCR amplicon and cleavage sizes after T7E1 assay.

Table S6. The mRNA scores for USP1 and MAST1 expression derived from the Cancer Cell Line Encyclopedia database.

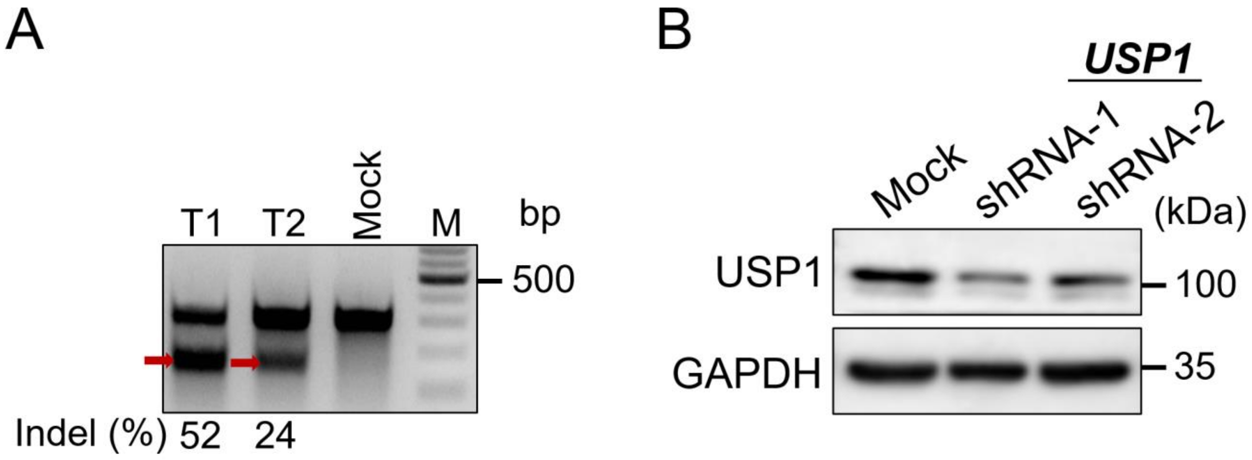

Figure S1. The efficiencies of sgRNAs and shRNAs targeting *USP1*.

(A) The cleavage efficiency of sgRNA1 (T1) and sgRNA2 (T2) was determined by the T7E1 assay in HeLa cells after transfection with plasmids encoding Cas9 and sgRNA targeting *USP1*. Scrambled sgRNA transfected cells were used as mock control (Mock). The size marker (M) is shown. The red arrow indicates the expected position of the cleaved DNA bands. The numbers at the bottom of the gel indicate the mutation percentages measured by band intensity using ImageJ software. (B) The efficiency of shRNAs was validated in HeLa cells by transducing shRNA1 and shRNA2 targeting *USP1* and immunoblotting with the USP1 antibody. GAPDH was used as a loading control.

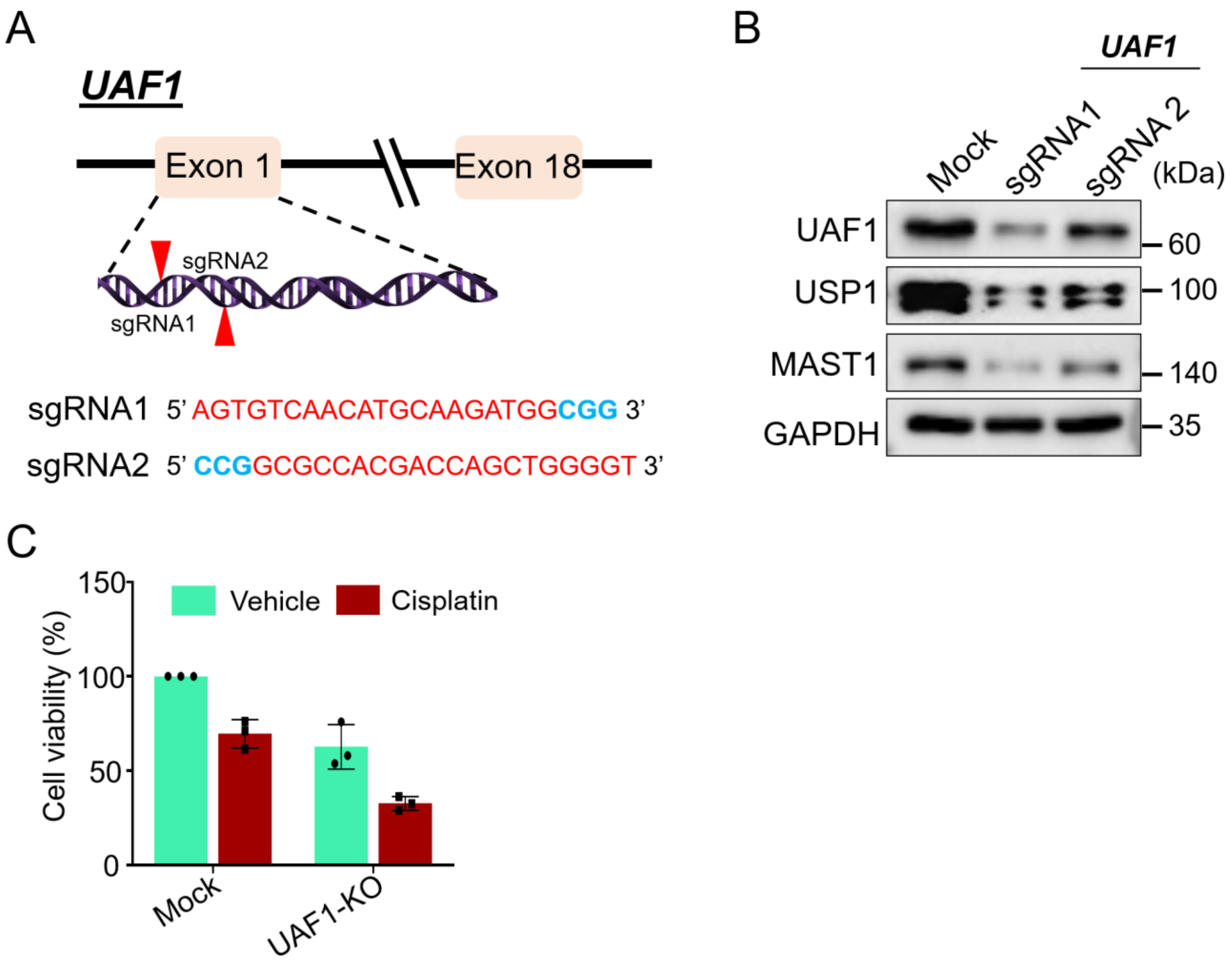

Figure S2. The effect of *UAF1* depletion on MAST1 protein level and cisplatin resistance.

(A) Schematic representation of the sgRNA targeting exon 1 of the *UAF1* gene. Red arrowheads indicate the positions of sgRNAs that target the top strand and the bottom strand of *UAF1* gene. sgRNA sequences are in red; PAM sequences are in bold blue font. (B) Validation of sgRNAs efficiency targeting *UAF1* and their effect on USP1 and MAST1 by transient transfection of sgRNAs into HeLa cells and immunoblotting with indicated endogenous antibodies. (C) The *UAF1*-depleted cells were treated with a sub-lethal dose of cisplatin for 48 h, and cell viability was assayed using CCK-8 reagent. Data are presented as the mean and standard deviation of three independent experiments (n = 3).

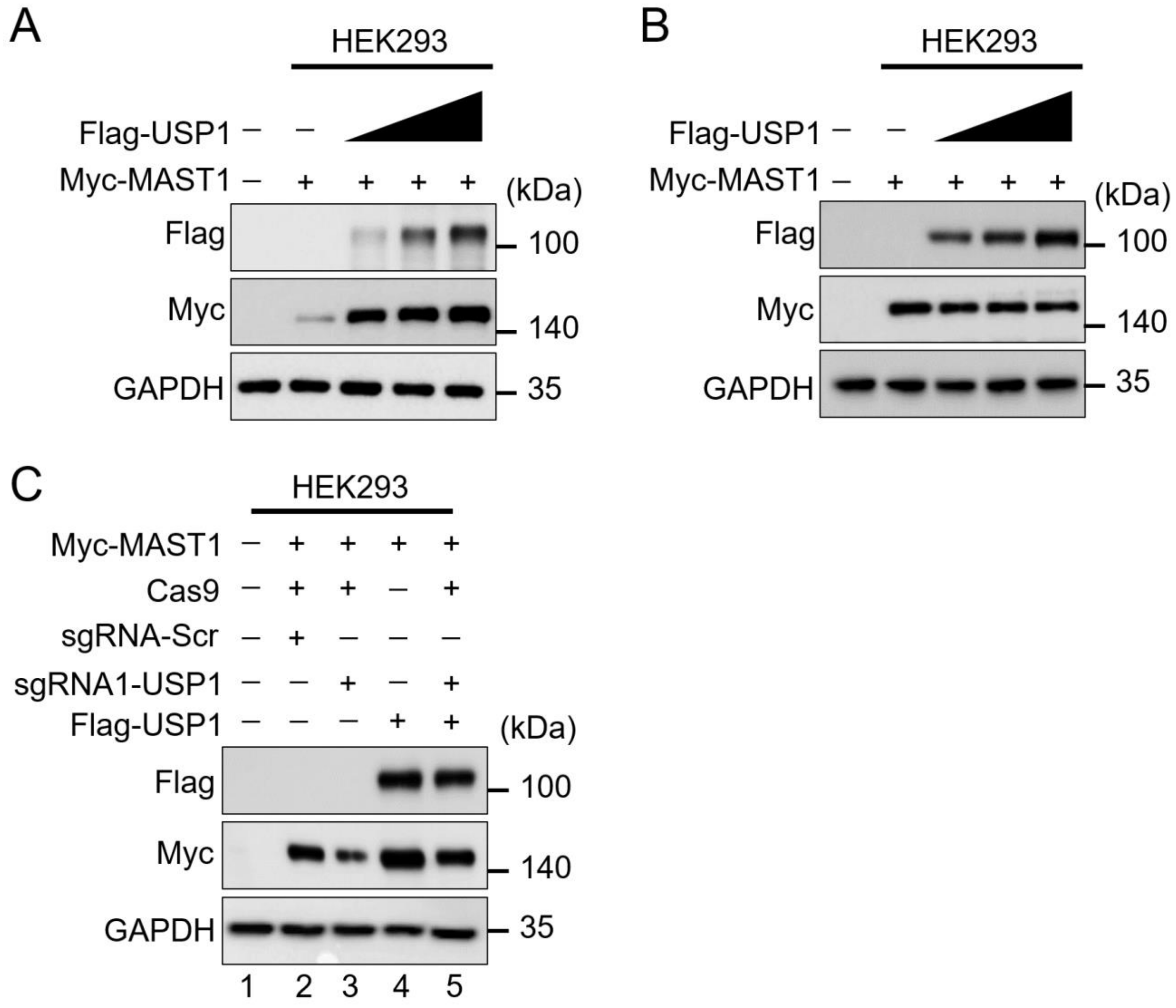

Figure S3. USP1 increases exogenous MAST1 protein level.

(A) Exogenous protein levels of MAST1 in HEK293 cells were analyzed upon transfection with increasing concentrations of Flag-USP1 or (B) USP1CS. (C) The reconstitution effect of USP1 on exogenous MAST1 protein in *USP1*-depleted HEK293 cells.

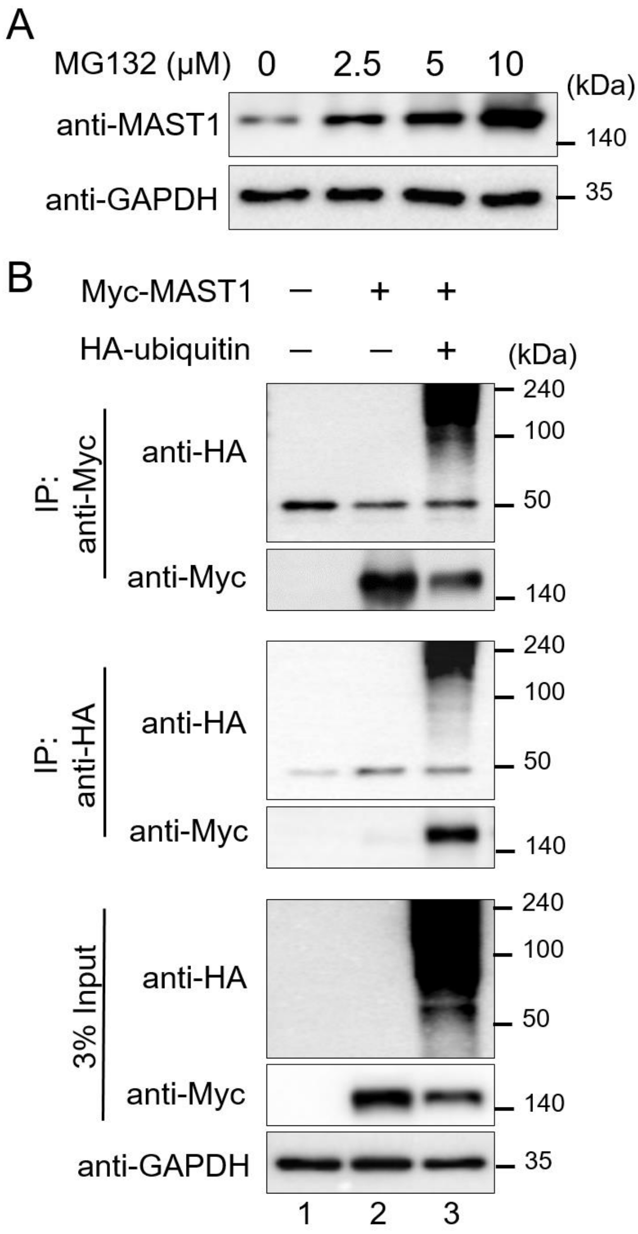

Figure S4. MAST1 protein undergoes 26S proteasomal degradation. (A) The MAST1 protein level was analyzed in HeLa cells treated with increasing concentrations of MG132 (0, 2.5, 5, and 10 μM/mL). (B) HEK293 cells were transfected with Myc-MAST1 and HA-ubiquitin to analyze the ubiquitination status of MAST1 protein. The protein obtained from the transfected samples were immunoprecipitated and immunoblotted with the indicated antibodies.

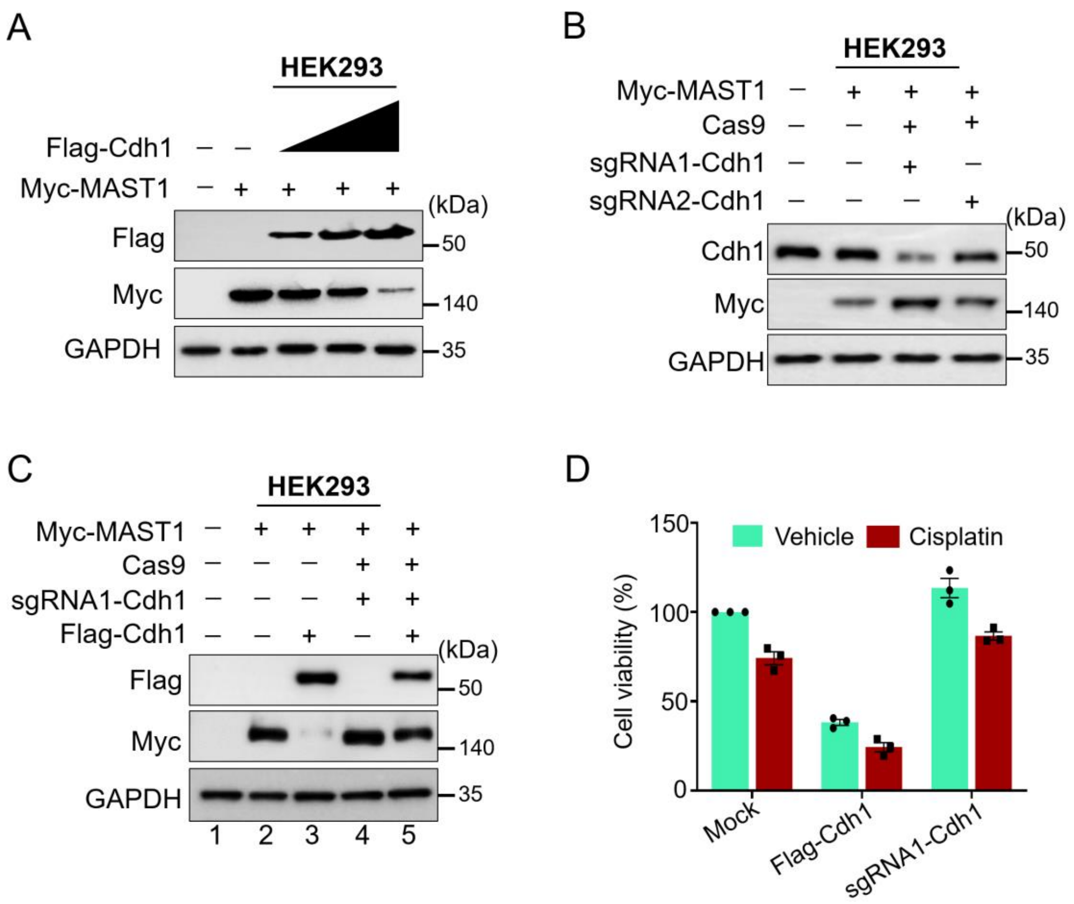

Figure S5. The effect of Cdh1 on MAST1 protein level and cisplatin resistance.

(A) Exogenous protein levels of MAST1 in HEK293 cells were analyzed upon transfection with a constant amount of Myc-MAST1 and increasing concentrations of Flag-Cdh1 (B) The exogenous protein level of MAST1 in HEK293 cells was analyzed upon transfection of sgRNA1 and sgRNA2 targeting *Cdh1*. (C) The Cdh1-mediated degradation of exogenous MAST1 protein was rescued in cells transfected with sgRNA1 targeting *Cdh1*. Protein expression was assessed by Western blotting with the indicated antibodies. GAPDH was used as a loading control. (D) The Cdh1-overexpressed and *Cdh1*-depleted cells were treated with a sub-lethal dose of cisplatin for 48 h, and cell viability was assayed using CCK-8 reagent. Data are presented as the mean and standard deviation of three independent experiments (n = 3).

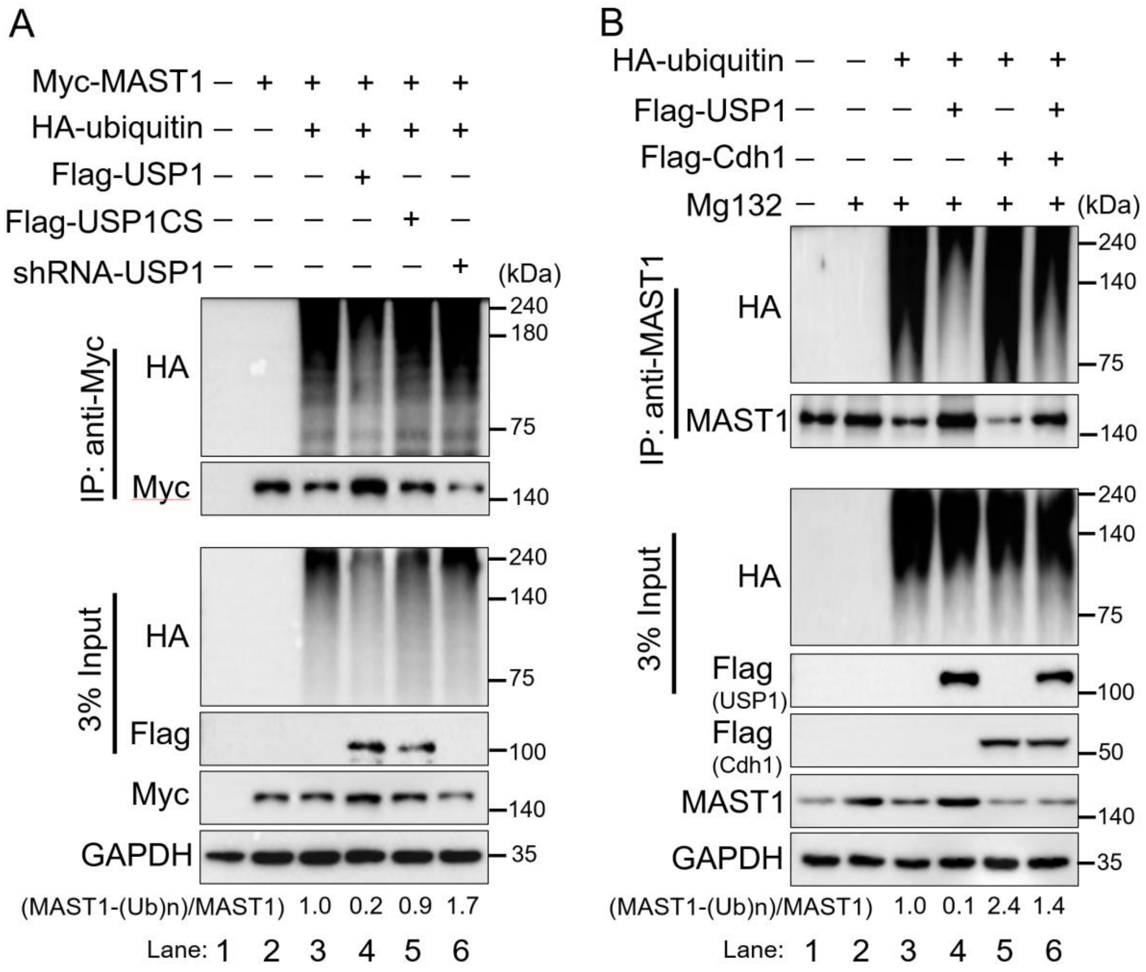

Figure S6. USP1 deubiquitinates Cdh1-mediated MAST1 ubiquitination.

(A) HEK293 cells were transfected with Myc-MAST1, HA-ubiquitin, Flag-USP1, Flag-USP1CS, or shRNA targeting *USP1*. The deubiquitination of exogenous MAST1 was confirmed by co-immunoprecipitation with the anti-Myc antibody and immunoblotting with the anti-HA and anti-Myc antibodies. (B) The deubiquitinating activity of USP1 was analyzed in the presence of Cdh1 by co-immunoprecipitation with the anti-MAST1 antibody and immunoblotting with the anti-HA and anti-MAST1 antibodies. GAPDH was used as the internal loading control. The ubiquitination of MAST1 was quantified using ImageJ software with reference to the input MAST1 and represented as (MAST1-(Ub)n/MAST1) below the blot.

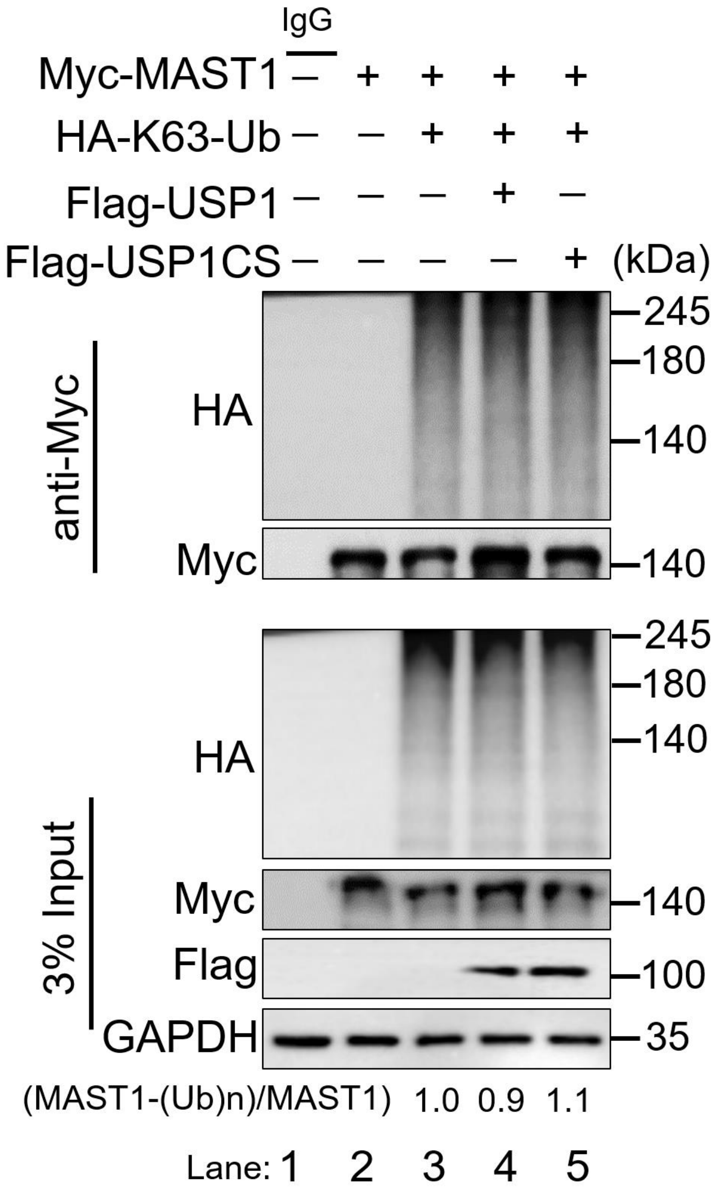

Figure S7. The deubiquitination of K63-linked ubiquitination of MAST1 by USP1. HEK293 cells were transfected with Myc-MAST1 and HA-K63-ubiquitin along with Flag-USP1 or Flag-USP1CS, followed by immunoprecipitation with an anti-Myc antibody and immunoblotting with anti-HA and anti-Myc antibodies. The ubiquitination of MAST1 was quantified using ImageJ software with reference to the input MAST1 and represented as (MAST1-(Ub)n/MAST1) below the blot.

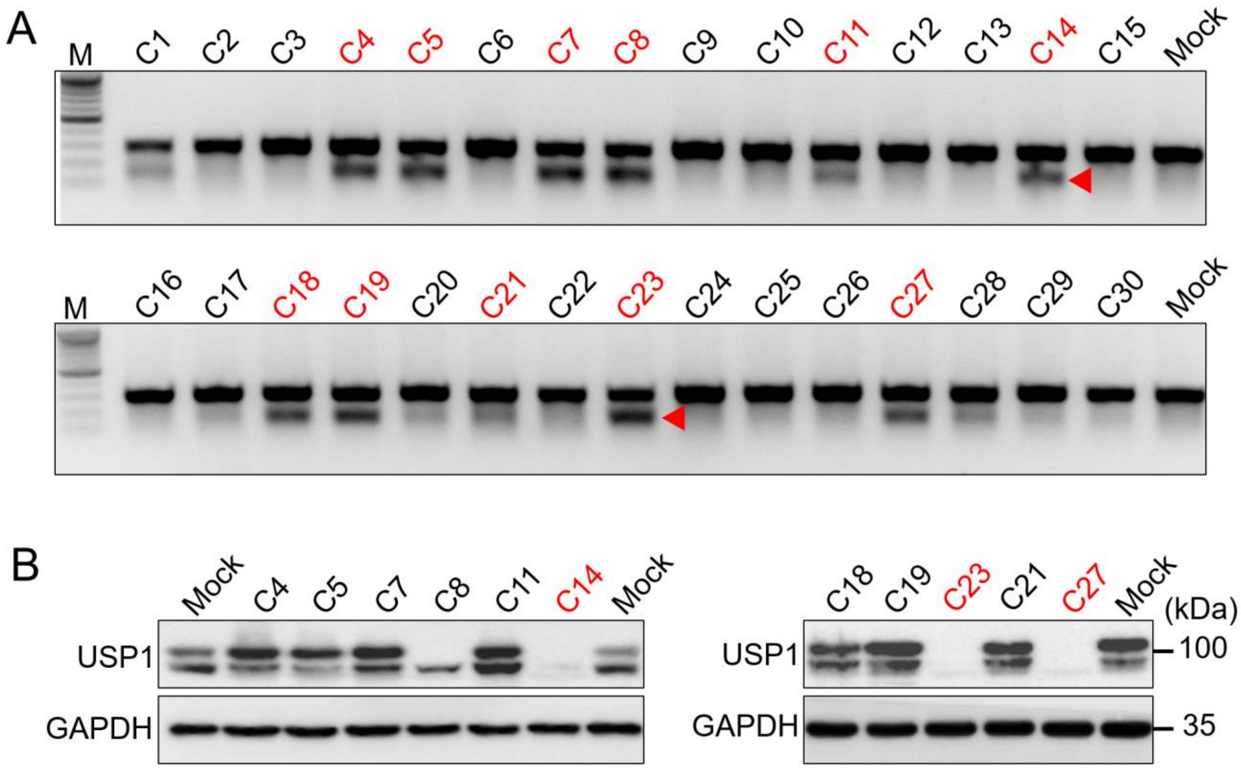

Figure S8. Generation of single cell-derived *USP1* knockout clones in A549 cells. (A) Screening for single cell-derived *USP1* knockout clones in A549 cells by T7E1 assay. T7E1 positive clones showing cleavage are represented in red text. Red arrow heads show the cleaved bands. (B) Western blot analysis showing the knockout efficiency of *USP1* in A549 cells. Scrambled sgRNA transfected cells were used as mock control (Mock).

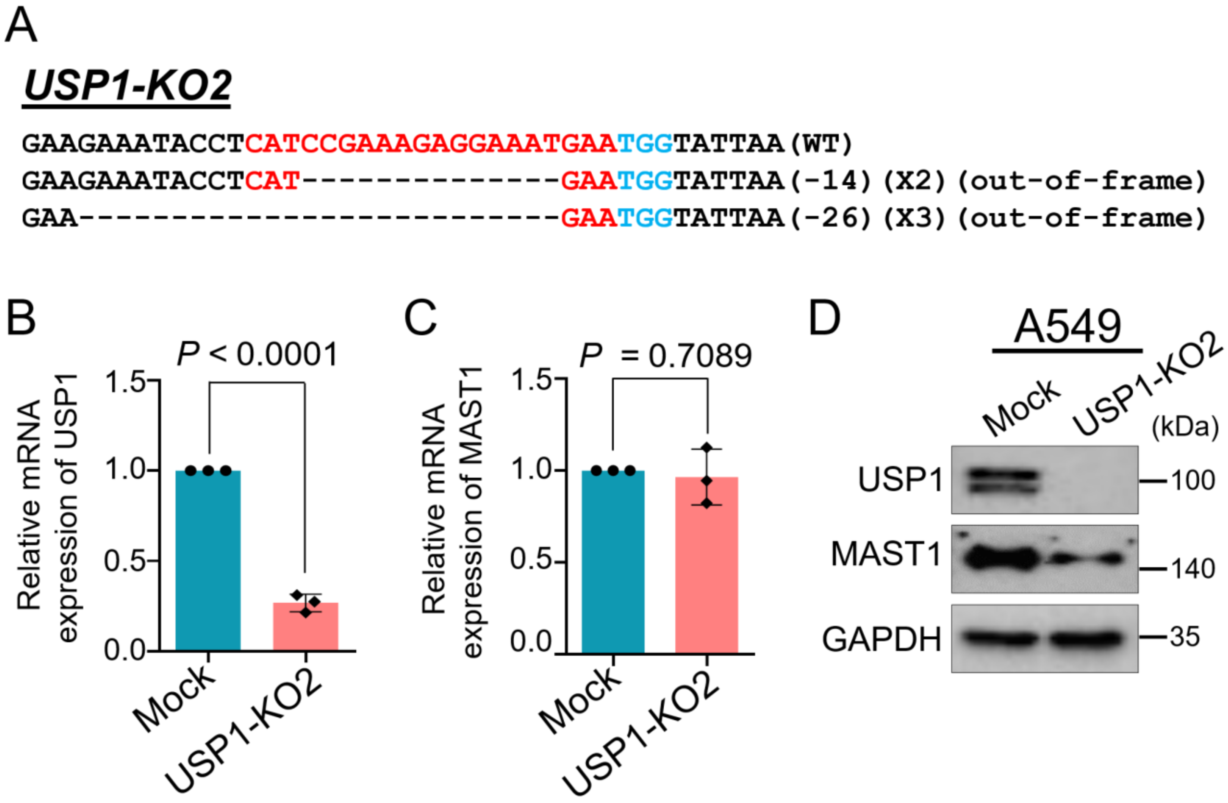

Figure S9. Validation of single cell-derived USP1-KO2 clone in A549 cells. (A) Sanger sequencing data showing the disrupted *USP1* gene sequences in USP1KO#23 (USP1-KO2). The sgRNA recognition site is denoted in red. The deleted bases are indicated with dashes and the number of deleted bases are indicated in parentheses. The number of occurrences of the indicated sequences is shown in parentheses (for example, X2 and X3 indicate the number of each clone sequenced). (B) The effect of USP1-KO2 on the mRNA expression of *USP1* and (C) *MAST1* was analyzed by qRT-PCR with specific primers. The relative mRNA expression levels are shown after normalization to GAPDH mRNA expression. Data are presented as the mean and standard deviation of three independent experiments (n = 3). A two-tailed *t*-test was used, and the *P* values are indicated. (D) Western blot analysis of the endogenous expression of USP1 and MAST1 protein in USP1-KO2. GAPDH was used as the internal loading control.

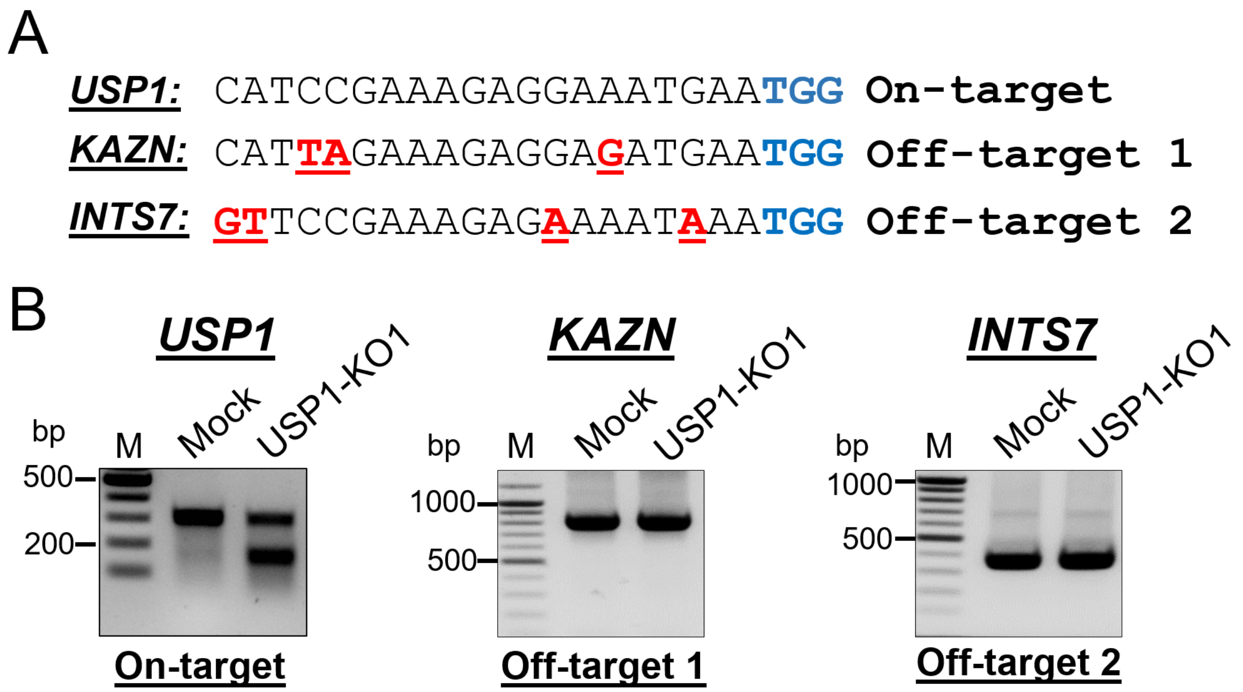

Figure S10. Off-target analysis of sgRNA1-targeting *USP1* in USP1 knockout clone.

(A) The on-target sequence of USP1, off-target 1 sequence of KAZN and off-target 2 sequence of INTS7. PAM sequences are shown in blue and mismatched bases are underlined and represented in bold red font. (B) The mutation frequencies at on-target and off-target sites were determined using T7E1 assays.

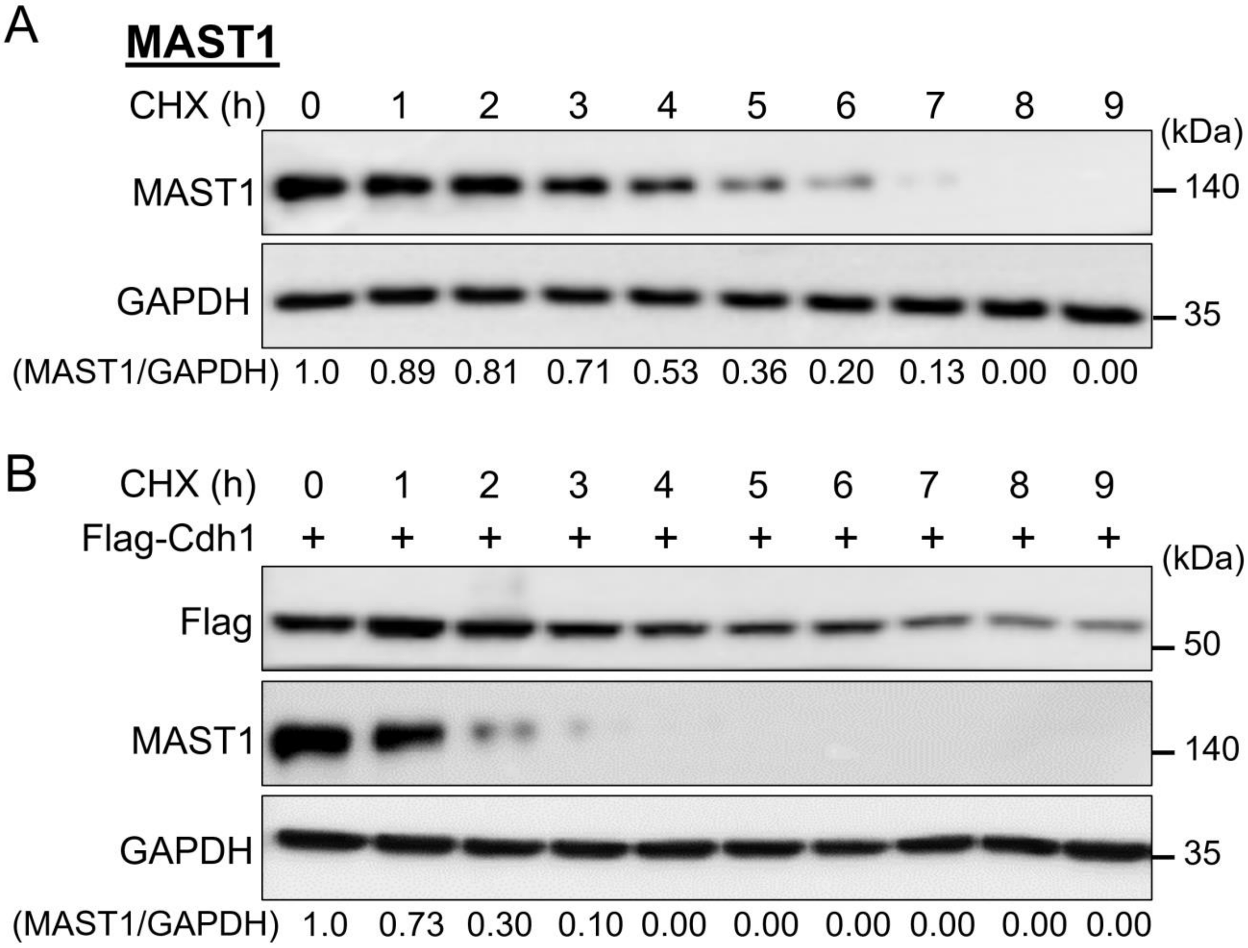

Figure S11. Half-life of MAST1 protein. (A) The half-life of MAST1 protein was determined using cycloheximide assay in HeLa cells for the indicated time intervals as mentioned above the blot. The rate of MAST1 decay was quantified using ImageJ software with reference to the GAPDH as control and is mentioned below the blot. (B) The effect of Cdh1 on the half-life of MAST1 protein was determined in HeLa cells transfected with Flag-Cdh1 and treated with CHX for the indicated time points. The rate of MAST1 decay was quantified using ImageJ software with reference to the GAPDH as control and is mentioned below the blot.

USP1

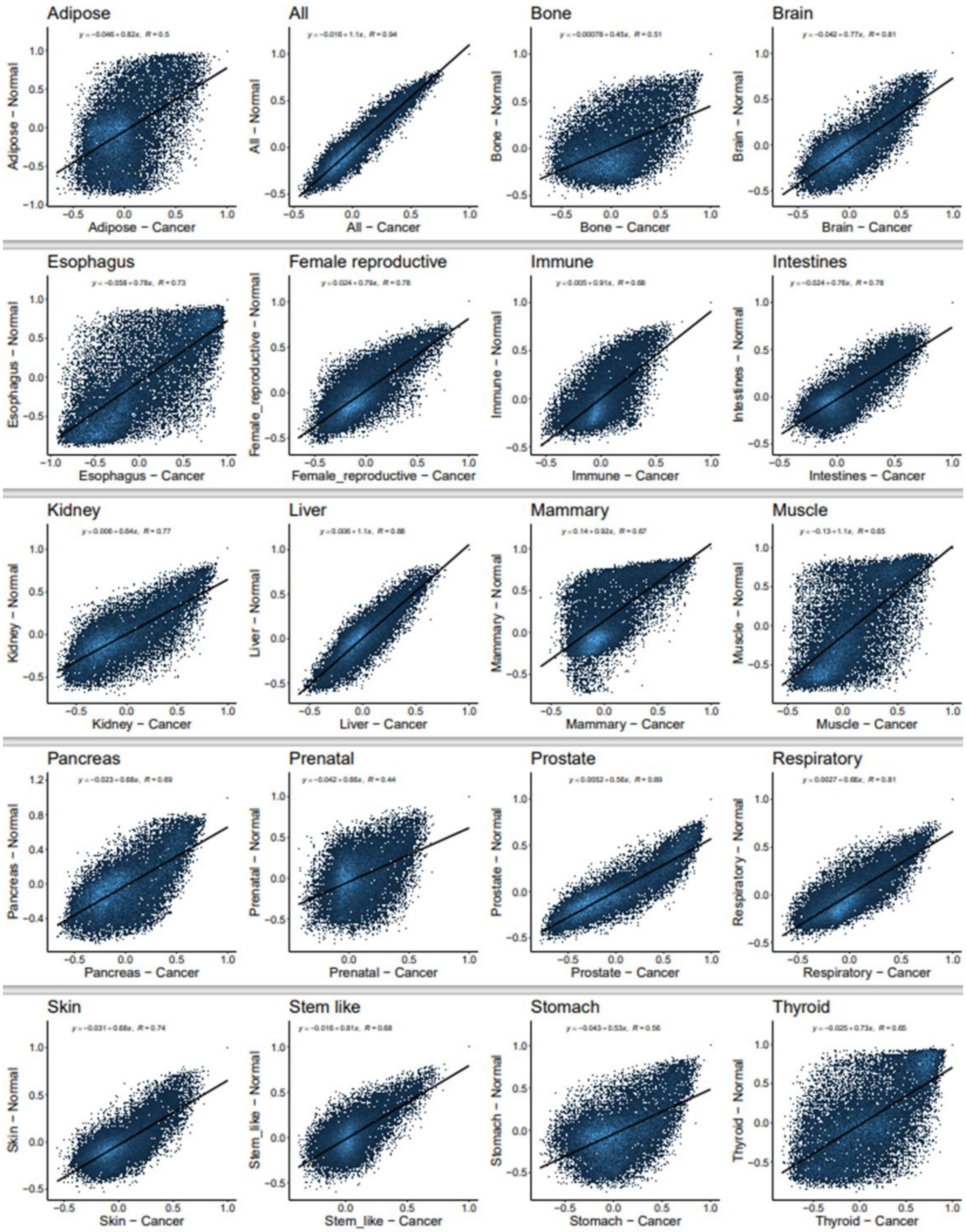

Adipose
Adipose − Normal
Adipose − Cancer
All
All − Normal
All − Cancer
Bone
Bone − Normal
Bone − Cancer
Brain
Brain − Normal
Brain − Cancer
Esophagus
Esophagus − Normal
Esophagus − Cancer
Female reproductive
Female_reproductive − Normal
Female_reproductive − Cancer
Immune
Immune − Normal
Immune − Cancer
Intestines
Intestines − Normal
Intestines − Cancer
Kidney
Kidney − Normal
Kidney − Cancer
Liver
Liver − Normal
Liver − Cancer
Mammary
Mammary − Normal
Mammary − Cancer
Muscle
Muscle − Normal
Muscle − Cancer
Pancreas
Pancreas − Normal
Pancreas − Cancer
Prenatal
Prenatal − Normal
Prenatal − Cancer
Prostate
Prostate − Normal
Prostate − Cancer
Respiratory
Respiratory − Normal
Respiratory − Cancer
Skin
Skin − Normal
Skin − Cancer
Stem like
Stem_like − Normal
Stem_like − Cancer
Stomach
Stomach − Normal
Stomach − Cancer
Thyroid
Thyroid − Normal
Thyroid − Cancer

Figure S12. Expression of USP1 in normal vs. cancer tissues. Scatter plot showing the expression of USP1 in different types of cancer tissues vs. normal contexts using Correlation AnalyzeR. The displayed *R* value was determined by Pearson correlation.

MAST1

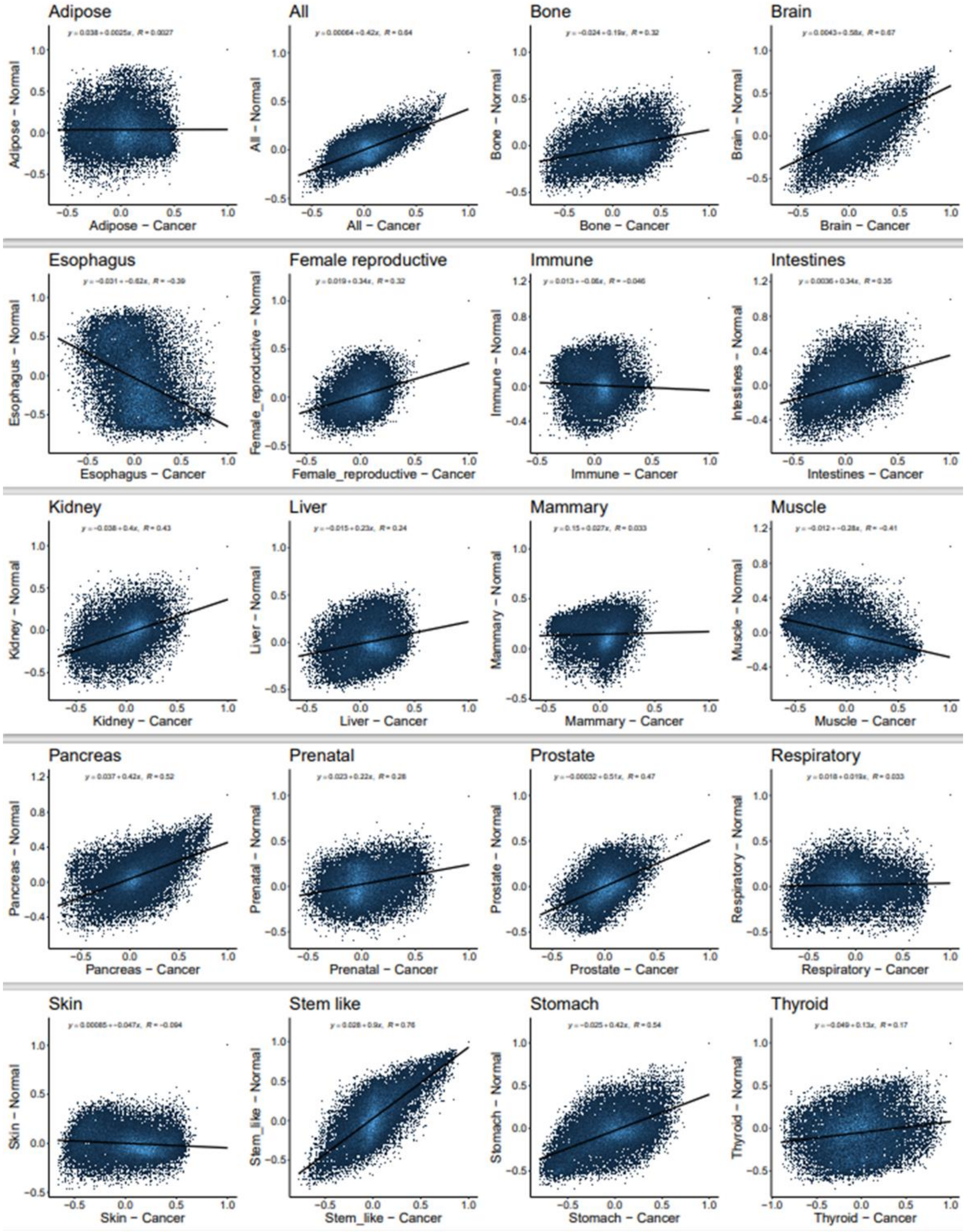

Adipose
Adipose - Normal
Adipose - Cancer
All
All - Normal
All - Cancer
Bone
Bone - Normal
Bone - Cancer
Brain
Brain - Normal
Brain - Cancer
Esophagus
Esophagus - Normal
Esophagus - Cancer
Female reproductive
Female_reproductive - Normal
Female_reproductive - Cancer
Immune
Immune - Normal
Immune - Cancer
Intestines
Intestines - Normal
Intestines - Cancer
Kidney
Kidney - Normal
Kidney - Cancer
Liver
Liver - Normal
Liver - Cancer
Mammary
Mammary - Normal
Mammary - Cancer
Muscle
Muscle - Normal
Muscle - Cancer
Pancreas
Pancreas - Normal
Pancreas - Cancer
Prenatal
Prenatal - Normal
Prenatal - Cancer
Prostate
Prostate - Normal
Prostate - Cancer
Respiratory
Respiratory - Normal
Respiratory - Cancer
Skin
Skin - Normal
Skin - Cancer
Stem like
Stem_like - Normal
Stem_like - Cancer
Stomach
Stomach - Normal
Stomach - Cancer
Thyroid
Thyroid - Normal
Thyroid - Cancer

Figure S13. Expression of MAST1 in normal vs. cancer tissues. Scatter plot showing the expression of MAST1 in different types of cancer tissues vs. normal contexts using Correlation AnalyzeR. The displayed *R* value was determined by Pearson correlation.

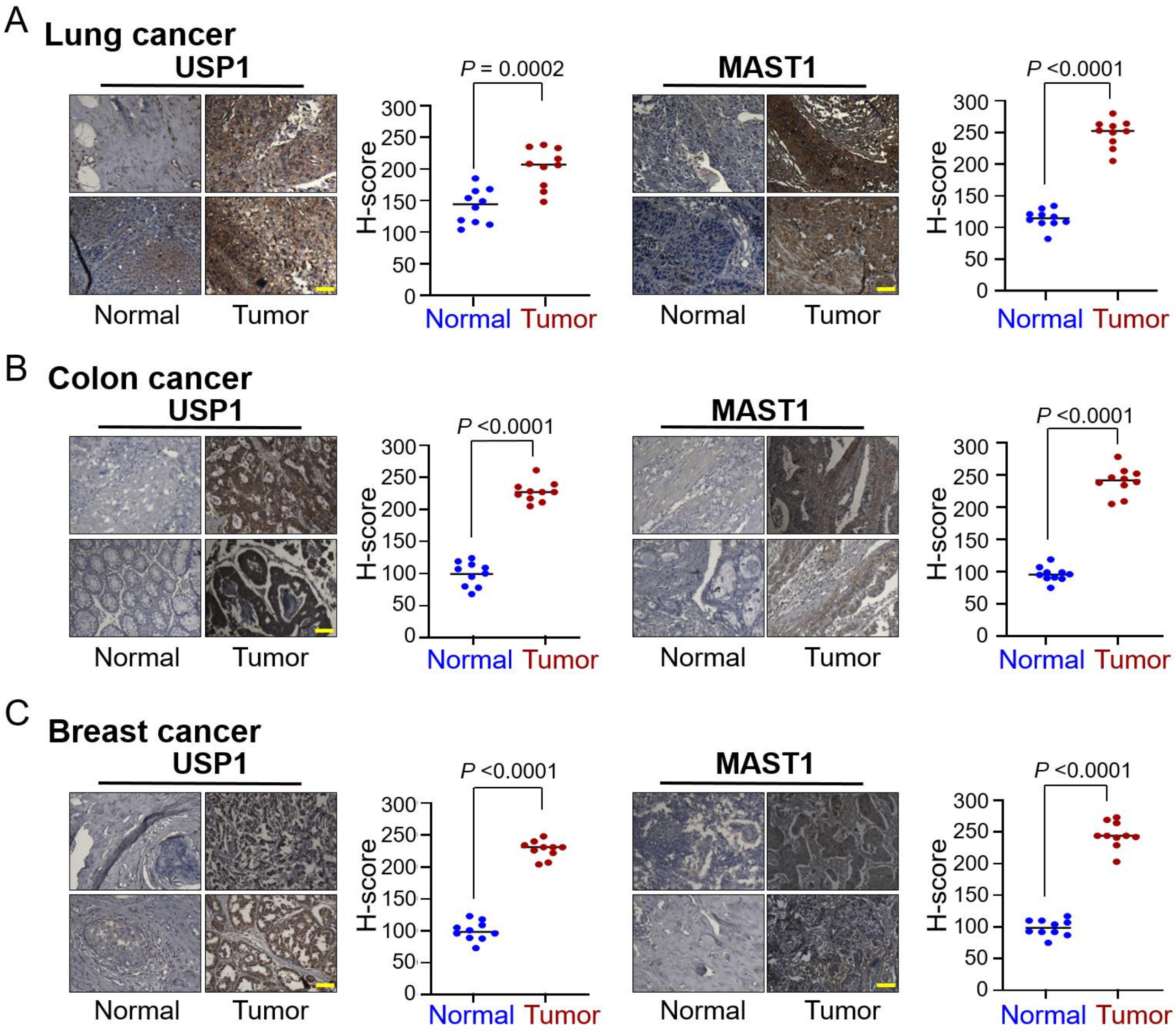

Figure S14. Immunohistochemical staining of USP1 and MAST1 in human clinical samples. Representative immunohistochemical staining images of endogenous USP1 and MAST1 in (A) lung, (B) colon, and (C) breast cancer tissues vs. normal tissues. All IHC images were quantified with an H-score. A two-tailed *t*-test was used, and *P* values are indicated. Scale bar = 30 µm.

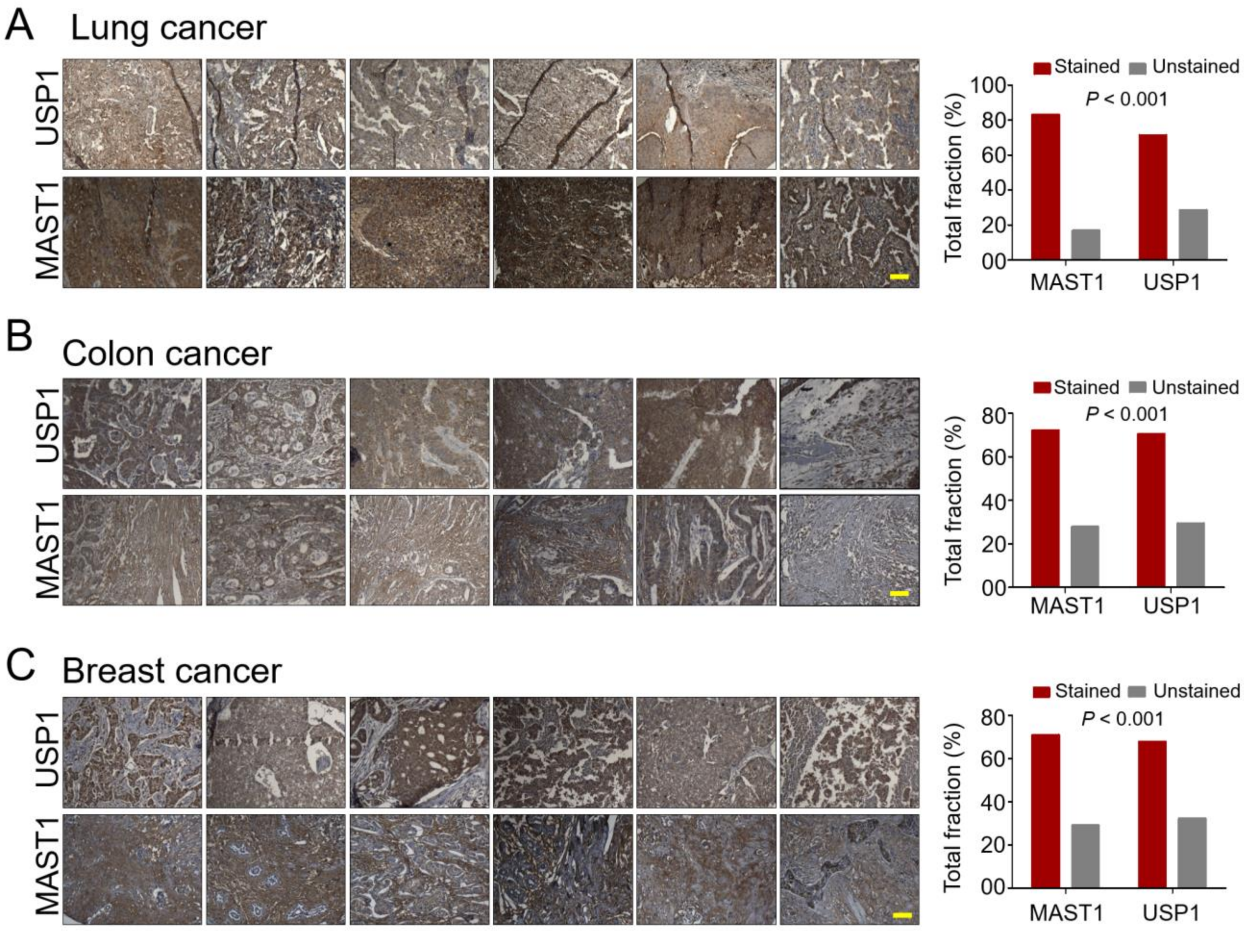

Figure S15. Immunohistochemical staining of USP1 and MAST1. Representative immunohistochemical (IHC) staining images of endogenous USP1 and MAST1 in (A) human lung cancer (n = 32), (B) colon cancer (n = 32), and (C) breast cancer (n = 21) tissues. The statistical significance of USP1 and MAST1 protein staining intensity was assessed using Chi-square test and the *P* values are indicated. Stained group represents highly stained cells and unstained group represents low or unstained cells.

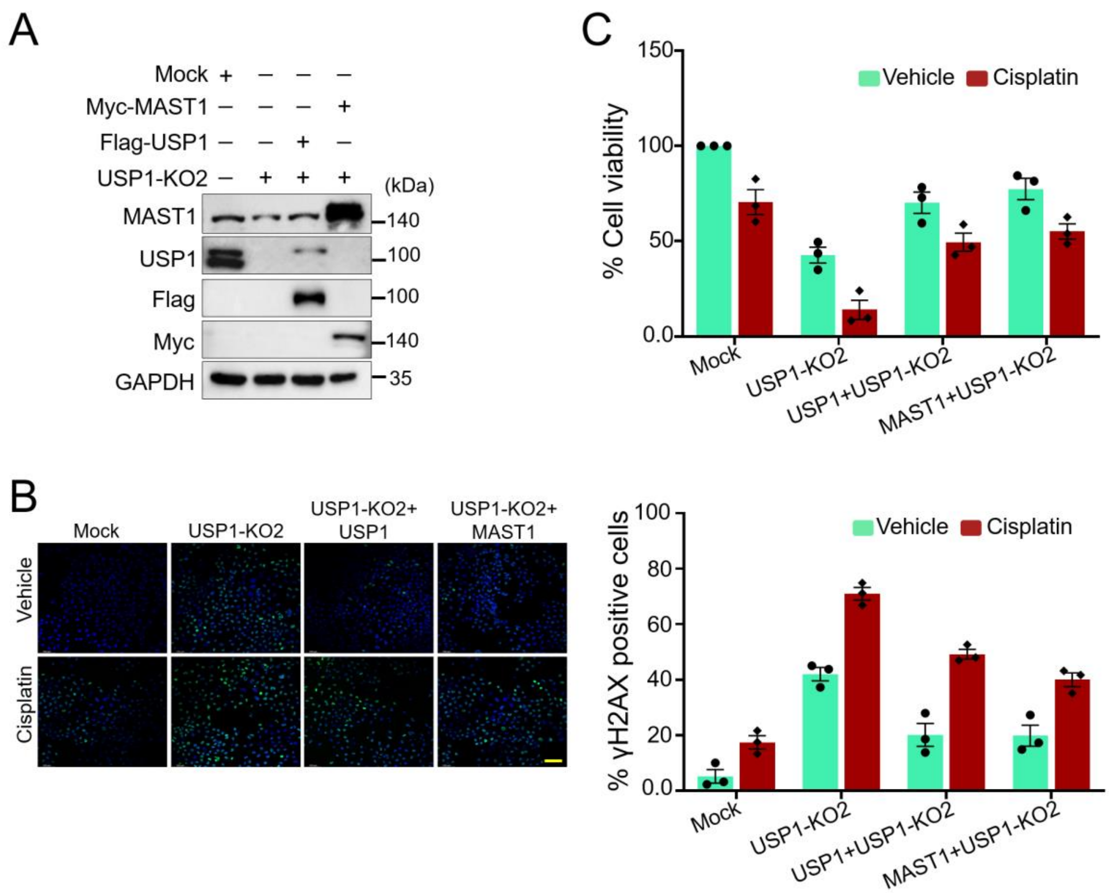

Figure S16. Depletion of USP1 promotes DNA damage and prevents cell proliferation. Mock control, USP1-KO2, and USP1-KO2 cells reconstituted with either USP1 or MAST1 were used to perform the following experiments. (A) Western blot analysis to validate the expression of USP1 and MAST1 using USP1- and MAST1-specific antibodies. GAPDH was used as the loading control. (B) The cells were treated with either vehicle or cisplatin (2 μg/mL) for 24 h and subjected to immunofluorescence analysis to estimate γH2AX foci formation. Green, γH2AX; blue, nucleus stained by DAPI. Scale bar = 100 μm. The right panel depicts the percentage of γH2AX-positive cells. (C) The cells were treated with a sub-lethal dose of cisplatin (2 μg/mL) for 48 h, and cell viability was assayed using CCK-8 reagent. Data are presented as the mean and standard deviation of three independent experiments (n = 3).

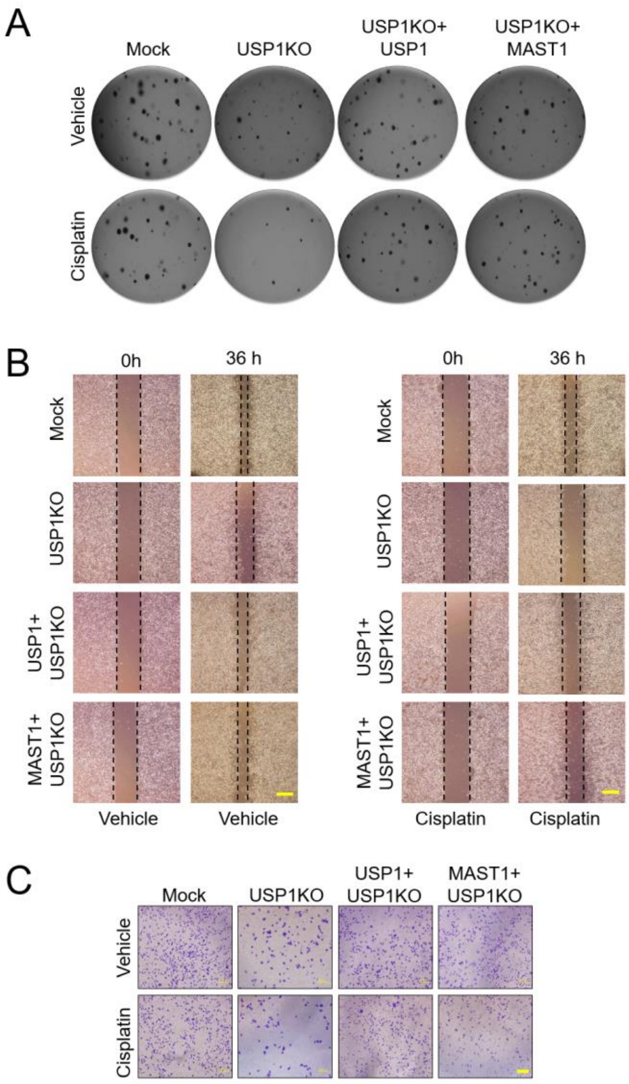

Figure S17. Determination of carcinogenic activity of USP1-KO1 in A549 cells. (A) Mock A549, USP1-KO1 A549, USP1-KO1_reconsUSP1 (USP1-KO1+USP1), and USP1-KO1_reconsMAST1 (USP1-KO1+MAST1) cells were treated with either vehicle or cisplatin for 14 days, and the colonies were stained with crystal violet for visualization. (B) Representative images of *in vitro* scratch assay to assess the migration potential of the groups mentioned. Scale bar = 100 μM. (C) Transwell cell invasion assay was performed with the groups mentioned. Scale bar = 500 μM. All assays were performed in triplicate and representative images are presented.

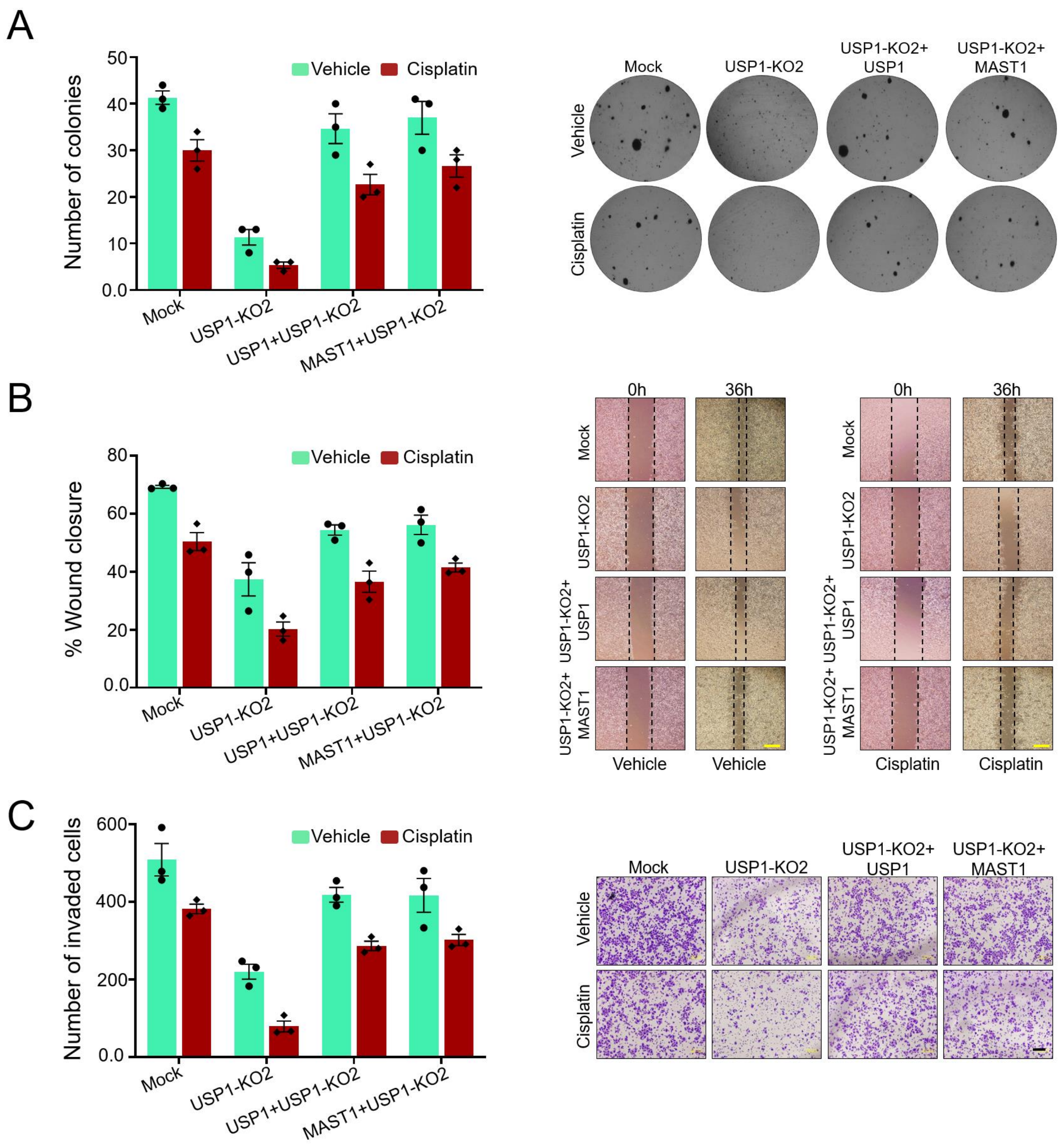

Figure S18. Determination of carcinogenic activity of USP1-KO2 in A549 cells. Vehicle- or cisplatin-treated, Mock A549, USP1-KO2 A549, USP1-KO2_reconsUSP1 (USP1-KO2+USP1), and USP1-KO2_reconsMAST1 (USP1-KO2+MAST1) cells were subjected to a (A) colony formation assay, the representative images are presented in the

right panel; (B) wound-healing assay, the representative images are presented in the right panel, Scale bar = 100 μM; (C) Transwell cell-invasion assay, the representative images are presented in the right panel, Scale bar = 500 μM. Data are presented as the mean and standard deviation of three independent experiments (n = 3).

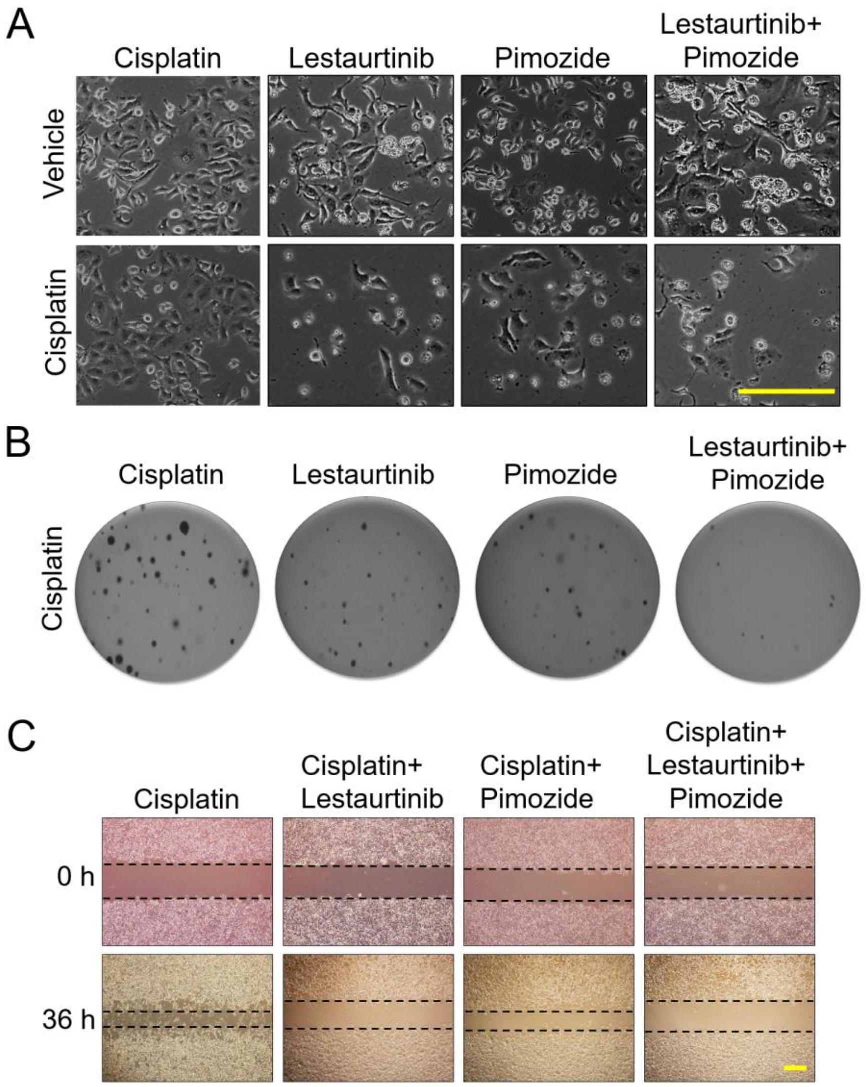

Figure S19. Combined pharmacologic inhibition of USP1 and MAST1 further sensitizes cisplatin-resistant cells to cisplatin. (A) The morphology of A549-cis^R cells subjected to single treatment of lestaurtinib (150 nM) or pimozide (25 μM) or their combination in presence of cisplatin (5 μg/mL) was assessed and representative images are presented. Scale bar = 200 μm. (B) The cells were treated with either vehicle or mentioned drugs for 14 days, and the colonies were stained with crystal violet for visualization. (C) Representative images of *in vitro* scratch assay to assess the migration potential of the groups mentioned. Scale bar = 100 μm.

Table S1. Target sequences used for sgRNA plasmid construction.

Gene	sgRNA	Direction	Sequence (5' to 3')	Orientation
USP1	sgRNA1	FP	CATCCGAAAGAGGAAATGAA	Sense
		RP	TTCATTTCCTCTTTCGGATG	
	sgRNA2	FP	GCATAGAGATGGACAGTATG	Sense
		RP	CATACTGTCCATCTCTATGC	
Cdh1	sgRNA1	FP	GCAGTACACGGAGCACCTGG	Sense
		RP	CCAGGTGCTCCGTGTACTGC	
	sgRNA2	FP	CGCTTCTGGAACACGCTGAC	Sense
		RP	GTCAGCGTGTTCCAGAAGCG	
UAF1	sgRNA1	FP	AGTGTCAACATGCAAGATGG	Sense
		RP	CCATCTTGCATGTTGACACT	
	sgRNA2	FP	GCGCCACGACCAGCTGGGGT	Antisense
		RP	ACCCCAGCTGGTCGTGGCGC	

Table S2. Target sequences used for shRNA plasmid construction.

Gene	shRNA	Sequence (5’ to 3’)
USP1	shRNA1	GAAAGCTCCACATCAATAA
	shRNA2	AGTGACCAAACAGGCATTA

Table S3. Oligonucleotide sequences used to get PCR amplicon for T7E1 assay.

Gene	sgRNA	Direction	Sequence (5’ to 3’)
USP1	sgRNA1 and sgRNA2	FP	AAACCTGGTGTATGGTAAGCA
		FP1	CAGCATGATGCACAGGAAGT
		RP	AGGAACACCAGTCATTGGAAGA
Cdh1	sgRNA1 and sgRNA2	FP	AACGACAACAAGGTACCCCC
		FP1	GCTGCTGGTCTGGAATCACT
		RP	CTATGCCTGCTGCCTCACAT
		RP1	CCATCTCCCTTCAGAGCGAC
UAF1	sgRNA1 and sgRNA2	FP	TGAGGCCACGACATAATTCTCC
		FP1	CAGCCCTGAAAAGTGGCTC
		RP	CCGCAAGTCCACTCCTACAGA

Table S4. Oligonucleotide sequences used to get PCR amplicon for Off-target genes.

Gene	Direction	Sequence (5' to 3')
KAZN	FP	TATGCTCTGCCTTGCATCGT
	FP1	TCACATAGCCTGCAGCACAA
	RP	TTCTTCCATCTGTGTATTCACTTGT
INTS7	FP	ATGTTCTGAGGCAACCTGAGT
	FP1	GAATAGAAGGAGCACACGGT
	RP1	CAACTATGCCTTCTCCCCTGG
	RP	AATGGGGGAGAAGCACACAC

Table S5. PCR amplicon and cleavage sizes after T7E1 assay.

Gene	sgRNA	PCR size	Cleavage size
USP1	sgRNA1	296	140+156
	sgRNA2	296	171+125
Cdh1	sgRNA1	355	299+56
	sgRNA2	355	151+196
UAF1	sgRNA1	564	238+326
	sgRNA2	564	362+202
KAZN	off-target for USP1	770	245+525
INTS7	off-target for USP1	348	219+129

Table S6. The mRNA scores for USP1 and MAST1 expression derived from the Cancer Cell Line Encyclopedia database.

Cell line	USP1 expression	MAST1 expression
SCLC21H	5.53169336	6.72955277
DMS79	6.82946905	6.348197
KPNRTBM1	4.68818036	6.31705076
NCIH1836	6.21742461	6.02147973
MHHNB11	4.95093493	5.94696474
NCIH1105	7.03562391	5.88044065
NCIH1963	6.31578358	5.86913112
NCIH2106	6.78345665	5.86071455
NCIH1694	7.29167722	5.85324717
NH6	6.13442632	5.77610399
CORL279	6.79506558	5.71671659
KPNYN	5.05093697	5.6363346
NCIH1092	5.87479697	5.63575439
LU135	7.91043287	5.63575439
NCIH1618	6.52073653	5.5649878
NCIH82	6.15542543	5.53418614
NCIH889	6.08342614	5.47702965
NCIH2171	6.78920758	5.47540919
NCIH69	5.93969674	5.4502215
CORL24	7.52034367	5.44327511
KELLY	5.59275601	5.41650199
NCIH446	5.83035675	5.37364821
NCIH146	6.17831627	5.28392177
CHP126	5.03606369	5.22958792
CORL47	6.23859581	5.22265002
NCIH2081	6.05961486	5.21101219
MDAMB134VI	5.26940724	5.16309614
SKNFI	5.40973035	5.14282184
SKNBE2	4.6467387	5.10391721
NCIH1436	6.55842071	5.09887429
NCIH2227	6.12205145	5.02812708
HCC33	7.27052894	4.94438996
SIMA	4.57288967	4.94063661
NCIH1184	6.21218021	4.93545975
NCIH1930	6.61676937	4.90496572
NMB	6.24849689	4.90303827

NCIH209	6.38594866	4.79597469
IMR32	6.19239146	4.77505054
NCIH524	6.42676793	4.75435307
NCIH1155	6.5147535	4.71699089
NCIH1876	7.12298325	4.70763522
NCIH1385	4.63517395	4.70708299
NCIH2196	6.10538475	4.69153417
NCIH1341	6.52700776	4.60525726
NCIH510	6.32930303	4.59514557
CORL311	5.88386516	4.44228004
COGN305	4.61588707	4.40190347
CORL95	6.32966182	4.40053793
DU4475	6.07274895	4.39780296
SKNDZ	6.11270013	4.34411834
DMS153	6.76937505	4.32048468
GOTO	5.47540919	4.29278175
SCLC22H	6.65663937	4.28835856
NB1643	5.48058827	4.26978124
TGW	6.2212971	4.22110373
SBC5	4.3262497	4.1473067
NCIH2029	7.09476389	4.11436703
NCIH1048	5.99254186	4.04089243
SHP77	5.32300971	3.97819563
CHP212	4.70929064	3.96624587
NCIH660	6.9518675	3.92504997
NCIH526	6.09655704	3.90496572
CHLA15	5.63575439	3.87184365
LAN2	6.21723072	3.85698569
CORL23	4.19927972	3.84699469
NCIH2077	4.78240857	3.82781903
CORL88	6.50207596	3.82781903
NB1	5.65191275	3.79805052
NCIH841	5.27015514	3.77399633
HCSC1	5.37885838	3.76341157
LS	5.8242587	3.66902677
NCIH854	3.68929916	3.66106548
DMS273	5.58285704	3.65878273
NH12	4.97773785	3.6287736
NCIH810	5.55826763	3.56681515
DMS114	6.17632277	3.48284828
BEN	3.20633065	3.47118746

COLO668	6.90412278	3.44890095
HCC2157	5.54132898	3.41818995
SKNSH	5.01122726	3.39231742
MDAMB453	5.00270252	3.37573454
NCIH2444	4.35191096	3.34056227
HCC1395	5.23572706	3.3262497
SNU398	5.48574882	3.30597052
22RV1	5.61028666	3.29865832
A427	5.34695689	3.27649667
HCC1195	4.08831124	3.24792751
NGP	5.41481206	3.19219417
NCIH522	6.30359812	3.18586655
HCC1187	6.08937103	3.169925
ZR751	4.76181714	3.15704371
NCIH2286	5.82781903	3.15218342
HCC1833	5.07339182	3.15055968
CALU6	5.29167722	3.10768787
DMS53	6.20925839	3.10601324
NCIH358	5.51884983	3.09085343
NCIH650	4.62585493	3.08406427
HDQP1	4.58796499	3.08065766
NCIH1975	4.67242534	3.07382023
COGN278	5.75702325	3.06004738
JHH4	4.60347799	3.04788733
NCIH661	5.62029315	3.04614178
RERFLCAI	5.31867837	3.01077984
NCIH2087	5.12556845	3.0071955
HCC1806	3.84197312	3.00360224
LK2	5.41481206	2.98913901
NCIH727	5.03077747	2.98550043
NCIH2110	4.88606234	2.98185265
NCIH2052	4.97085365	2.97636364
HCC2108	5.138323	2.94110631
NCIH1793	4.53294029	2.94110631
NCIH838	5.46172499	2.93357264
NCIH1373	4.95046841	2.93168306
NCIH1581	5.1473067	2.91647664
LNCAPCLONEFGC	4.24488706	2.89724043
KPNSI9S	4.21179098	2.88752527
LXF289	4.42559358	2.88166462
HEP3B217	4.94204526	2.87774425

NCIH1703	5.47702965	2.86591882
LC1F	4.82680268	2.84398384
NCIH1299	5.20750246	2.83592407
SKMES1	4.93073734	2.76341157
DMS454	5.30451104	2.75274859
HCC1500	5.96439863	2.75060651
C33A	6.09360243	2.73552218
NCIH1819	5.04395687	2.72246602
NCIH1339	5.11228311	2.72246602
NCIH2126	5.13955135	2.71149491
MDAMB157	5.60050765	2.69599381
NCIH2030	4.70542504	2.68706069
EFM19	5.38404981	2.592158
MDAMB468	6.19672506	2.58976349
CAL51	6.50827018	2.57773093
BT549	5.94157586	2.57531233
HCC366	5.89384756	2.53853816
CALU1	6.24697806	2.52857132
SKNAS	4.86245139	2.52606881
KPL1	4.53915881	2.52356196
HCC1438	5.93262816	2.50842865
HCC1359	5.50493813	2.50334874
BOKU	4.90929309	2.50080205
NCIH2887	4.49952702	2.48284828
NCIH520	5.83238372	2.46988598
RERFLCMS	4.69766263	2.45154083
NCIH23	5.38059093	2.45154083
HS578T	4.45154083	2.44890095
MFM223	4.80115866	2.43562859
NCIH441	5.582556	2.42760617
NCIH2172	5.52199296	2.41953889
HCC1171	4.9044841	2.40599236
MDAMB436	5.83617663	2.40326772
NCIH1650	5.53791725	2.39231742
NCIH3255	4.71863562	2.38956681
T3M10	5.11686376	2.37851162
JHH2	5.64875303	2.37573454
HCC95	4.89530262	2.37016428
COLO824	7.56338697	2.37016428
CORL105	3.88849974	2.35614381
NCIH2085	5.34660238	2.33628339

IALM	4.70431868	2.29278175
VCAP	4.83541884	2.28392177
LCLC97TM1	4.34978987	2.28095631
MORCPR	4.79649393	2.27500705
HMC18	4.81915722	2.26303441
NCIH596	5.52638186	2.25701062
NCIH1838	4.74577515	2.25701062
HCC1599	5.8117279	2.25398927
NCIH2023	5.24450655	2.24184018
NCIH1573	4.9293176	2.24184018
EKVX	4.78293271	2.22342255
PC3	4.50462039	2.20476675
SW1271	5.74899785	2.1953476
T47D	4.33771109	2.17312743
NCIH2009	5.52418908	2.16349873
SUM1315MO2	6.21276386	2.16027483
CAMA1	4.48864354	2.15380534
NCIH1792	5.19219417	2.1473067
HCC202	4.50143915	2.14077866
HCC38	5.9044841	2.13750352
NCIH1734	5.69069644	2.12763328
MPP89	5.29020321	2.12432814
HCC364	5.49729301	2.12432814
NCIH2291	5.18110255	2.12432814
CAL120	5.64067876	2.12432814
EPLC272H	6.33181268	2.10097765
PC9	5.05701697	2.05311134
HCC2450	5.55305325	2.04963077
NCIH1437	4.25171909	2.04264434
CHAGOK1	5.38301302	2.01435529
LCLC103H	4.60644223	2.01077984
NCIH1355	5.74550627	2.0071955
BT483	4.1268077	2.0071955
SNU449	4.81455042	1.99638875
SNU182	4.40190347	1.99276843
CAL12T	4.90881291	1.97085365
NCIH1648	4.582556	1.96347412
NCIH1395	3.54101915	1.95605665
PLCPRF5	4.75221337	1.92599942
UACC893	4.53543092	1.92219785
NCIH1915	5.13791332	1.91838623

LU99	5.90881291	1.91838623
PC14	4.97773785	1.9068906
RERFLCKJ	6.14241344	1.88752527
HCC1937	5.26303441	1.87578006
LOUNH91	5.10055722	1.8559897
HCC1419	4.40463068	1.8559897
HCC827GR5	5.34837408	1.84398384
NCIH211	6.04264434	1.83187724
JHH5	4.83238372	1.81557543
CASKI	4.43429462	1.79077204
JHH1	4.38818954	1.79077204
HCC4006	4.6363346	1.78659636
SNU761	3.7473874	1.78659636
BT20	5.41616417	1.76977174
SKHEP1	4.79077204	1.75274859
SNU878	4.94063661	1.7398481
HCC1569	5.3248106	1.71808758
NCIH2342	5.25247621	1.71369582
JHH6	5.197315	1.70043972
NCIH2228	5.61735726	1.69599381
RERFLCAD2	4.49952702	1.68706069
DV90	4.64789009	1.67355642
NCIH1568	6.13257684	1.66448284
NCIH1693	6.12432814	1.65992456
MDAMB231	5.89893387	1.65535183
MSTO211H	5.53791725	1.63226822
HCC1954	5.5533605	1.60407132
HCC1143	5.60229059	1.59454855
NCIH2882	5.90616907	1.57046293
HCC461	5.92552477	1.55090067
HCS2	7.20035918	1.55090067
MCF7	4.28688115	1.54596837
SUM149PT	4.12018603	1.54596837
A549	4.87332106	1.53106949
NCIH2073	4.86245139	1.51601515
SW900	4.59753117	1.51601515
HCC44	5.00225245	1.50080205
SUM52PE	3.96254902	1.50080205
HCC1428	5.14363831	1.49057013
NCIH460	5.05701697	1.48542683
ACCMESO1	5.28651156	1.47508488

SW1573	5.51664556	1.47508488
NCIH1755	5.69988473	1.46988598
NCIH647	5.31795522	1.46988598
HELA	5.79155363	1.46988598
HCC827	5.39711841	1.45943162
NCIH1435	5.16430358	1.43295941
C4I	5.13134254	1.43295941
HCC2279	4.34340782	1.38404981
NCIH2347	5.28281044	1.36176836
LC1SQSF	5.86888427	1.36176836
NCIH2122	4.13750352	1.35614381
HCC78	4.27500705	1.35049725
AU565	5.01569381	1.35049725
SNU886	5.4551628	1.33342373
MDAMB175VII	3.57046293	1.31614574
DOTC24510	7.01870093	1.31034012
HEPG2	4.50652578	1.29865832
NCIH292	4.77978501	1.28095631
CAL148	4.88264305	1.23878686
SKBR3	4.99050111	1.23266076
GIMEN	4.26453643	1.23266076
HUH1	4.45351758	1.22650853
LUDLU1	4.53106949	1.22032996
P4E6	3.8509994	1.21412481
HCC15	5.78816366	1.20789285
NCIH1869	4.91838623	1.1953476
HOP92	3.84398384	1.16349873
HUH7	5.1886383	1.16349873
HCA1	5.02281165	1.11769504
HOP62	5.35155766	1.10433666
NCIH1623	5.29939121	1.0976108
HARA	5.52732061	1.09085343
NCIH1651	5.32156738	1.077243
MDAPCA2B	4.66789213	1.077243
HCC2218	3.65420638	1.06350294
LU65	4.83642913	1.05658353
SNU475	4.77663042	1.05658353
HCC2814	4.75648961	1.03562391
NCIH322	5.05180711	1.01435529
COLO699	5.48091135	0.99276843
NCIH2405	5.3950628	0.97819563

SQ1	5.53822774	0.97819563
CAL851	4.72955277	0.96347412
RERFLCAD1	5.17512535	0.95605665
HOKUG	6.2981084	0.95605665
EBC1	5.68201139	0.94860085
NCIH1781	5.21955577	0.93357264
HUH6	5.05961486	0.91838623
JHH7	5.23649262	0.91838623
RERFLCSQ1	5.20241772	0.91073266
HCC2935	4.47573343	0.89530262
MERO41	5.924575	0.88752527
MDAMB361	4.73714592	0.88752527
CALU3	4.54966917	0.87184365
JIMT1	6.15319733	0.83187724
SIHA	5.7057015	0.81557543
NCIH1563	4.94906797	0.80735492
SHMAC5	3.0976108	0.79077204
NCIH3122	5.29755828	0.78240857
VP229	3.99367436	0.78240857
SUM159PT	5.29682446	0.77399633
HCC2429	5.28281044	0.76553475
BPH1	6.08065766	0.74846123
MERO83	5.15583017	0.73118324
NCIH2170	4.38818954	0.72246602
JL1	4.28318098	0.72246602
MERO48A	5.5428771	0.72246602
ZR7530	4.6099913	0.70487196
HCC515	5.65935376	0.66902677
KNS62	3.89044669	0.64154603
BT474	4.89336221	0.63226822
LI7	4.79181407	0.63226822
NCIH1944	5.53884852	0.61353165
EFM192A	4.3262497	0.61353165
HT3	3.86195536	0.61353165
NCIH196	6.40684276	0.60407132
SW756	6.64500988	0.59454855
MERO82	5.72164608	0.56559718
SNU423	5.02147973	0.54596837
NCIH1666	4.17392693	0.50589093
ABC1	5.53915881	0.50589093
HCC70	6.65047737	0.50589093

SUM185PE	2.69376571	0.48542683
MS751	6.03738222	0.48542683
UACC812	3.16027483	0.43295941
MERO14	4.84749588	0.41142625
MDAMB415	4.20554891	0.40053793
SUM44PE	2.33913739	0.40053793
WPE1NA22	4.68706069	0.40053793
MERO84	4.98276546	0.38956681
SUM229PE	4.52293456	0.38956681
ISTMES1	5.21606678	0.37851162
HLF	5.83895177	0.37851162
ISTMES2	5.78371856	0.35614381
SISO	5.62117275	0.3219281
SNU387	5.627315	0.3219281
MERO95	5.05484848	0.27500705
SW954	1.94485845	0.21412481
NCIH28	5.67722633	0.20163386
NCIH226	5.25889576	0.18903382
C4II	6.06995989	0.18903382
SUM102PT	1.57046293	0.0976108
HCC1588	4.00809242	0.07038933
NCIH2452	5.67468662	0.05658353
SHMAC4	3.65878273	0.05658353
MERO25	6.55428186	0.04264434
